# Single-cell gain-of-function mapping reveals latent regulatory programs governing CD8^+^ T cell fate

**DOI:** 10.21203/rs.3.rs-9929244/v1

**Published:** 2026-06-11

**Authors:** Brandon M. Pratt, Genevieve N. Mullins, Nolan J. Brown, Lara van Rooyen, William D. Green, Fucong Xie, Vasyl Zhabotynsky, Jennifer Modliszewski, Nicholas de Vries, Alexander N. Jambor, Gabrielle Cannon, Jarred M. Green, Zaid Syed, Alec L. Plotkin, Andrew Kennedy, Coral Del Mar Alicea Paunto, Huitong Shi, Emily F. Merritt, Takeshi Egawa, Ashwin Somasundaram, Wei Wang, Jessica E. Thaxton, Gianpietro Dotti, H. Kay Chung, J. Justin Milner

**Affiliations:** 1 Lineberger Comprehensive Cancer Center, University of North Carolina at Chapel Hill, Chapel Hill, NC, USA; 2 Department of Pharmacology, University of North Carolina at Chapel Hill, Chapel Hill, NC, USA; 3 Department of Microbiology and Immunology, University of North Carolina at Chapel Hill, Chapel Hill, NC, USA; 4 Center for Nanotechnology in Drug Delivery, Eshelman School of Pharmacy, University of North Carolina at Chapel Hill, Chapel Hill, NC, USA; 5 Department of Chemistry and Biochemistry, University of California San Diego, La Jolla, CA, USA; 6 Curriculum in Bioinformatics and Computational Biology, University of North Carolina at Chapel Hill, Chapel Hill, NC, USA; 7 Department of Cell Biology and Physiology, University of North Carolina at Chapel Hill, Chapel Hill, NC, USA.; 8 Department of Pathology and Immunology, Washington University School of Medicine, St. Louis, MO, USA

## Abstract

CD8^+^ T cells mediate host defense and tumor immunity through specialized differentiation states, yet the regulatory programs that guide these states may also limit their functional potential. Loss-of-function studies have defined many regulators required for T cell differentiation, but they do not readily reveal regulatory activities that emerge only when transcription factors are ectopically expressed outside their native lineage, dosage, or temporal context. Here, we developed single-cell gain-of-function (GOF) sequencing (scGOF-seq), a multiplexed platform for *in vivo* mapping of transcription factor overexpression in antigen-specific CD8^+^ T cells across immunocompetent models of infection and cancer. By enforcing expression of canonical T cell regulators, lineage-silenced developmental factors, and temporally restricted transcription factors, scGOF-seq uncovered unexpected *in vivo* activities. Developmental regulators normally silenced in T cells, including NANOG, SOX2, OCT4 and GATA2, reshaped T cell differentiation in context-dependent ways, with NANOG promoting stemness-associated phenotypes and accumulation during chronic infection. In parallel, sustained cMyc expression outside its native temporal window generated a stem-like, effector-featured state with enhanced metabolic fitness, reduced terminal exhaustion, and profound antigen-dependent expansion exceeding 5,000-fold. Importantly, cMyc GOF maintained cell-cycle checkpoint signatures and demonstrated a strong dependence on antigen presence for proliferation across the tested conditions. scGOF-seq further identified cooperating transcription factor modules that complemented cMyc-driven programs and improved T cell responses in solid tumors. These findings establish systematic GOF perturbation as a framework for uncovering latent and temporally constrained regulatory activities in CD8^+^ T cells and guiding immune-state engineering.

Cell fate diversification is a defining feature of the immune system, with T cells adopting a spectrum of functionally specialized states across contexts such as infection and cancer^1-4^. These states are specified by transcription factor (TF) networks operating within tightly constrained regulatory systems shaped by signaling inputs, transient expression dynamics, and chromatin-encoded feedback^5-9^. Because T cell differentiation follows these restricted physiological trajectories, redirecting cellular programs toward novel, therapeutically effective states has become a central goal of cellular engineering. To date, most insights into T cell fate control have largely come from loss-of-function (LOF) approaches, including conditional knockouts, CRISPR-based perturbations, and RNA interference^9-15^. These studies have been instrumental in identifying essential regulators and enhancing T cell function^10,14,16,17^. However, because LOF approaches are designed to test the consequences of removing or disrupting endogenous regulators, they primarily define factors required for, or restraining, specific cell states. Complementary approaches are therefore needed to determine how enforced regulatory activity can expand, redirect, or reveal latent differentiation programs that remain otherwise inaccessible.

In parallel, the clinical success of adoptive T cell therapies in hematologic malignancies has expanded the genetic design space for therapeutic engineering^18,19^. Unlike endogenous immune responses, adoptive cell therapy permits extensive *ex vivo* manipulation, enabling gain-of-function (GOF) perturbations and synthetic biology strategies that circumvent physiological regulatory constraints. Recent GOF screens have begun to identify regulators that enhance T cell activity in cancer^20-28^, highlighting enforced gene activation as a complementary and largely untapped approach to T cell engineering. However, how GOF perturbations reshape the *in vivo* landscape of CD8^+^ T cell states, and whether they can systematically reveal latent or otherwise inaccessible differentiation programs with therapeutic potential, remains unclear.

Mechanistically, GOF perturbations can sustain TF activity beyond endogenous transcriptional and epigenetic constraints, enabling regulatory functions that may not be evident from endogenous expression patterns or LOF genetics. Individual GOF studies have illustrated this principle by identifying previously unappreciated regulators of T cell differentiation and function, such as LTBR^20,24,29^. More broadly, enforced TF activity can reveal regulatory biology through multiple mechanisms, including amplification of endogenous programs, extension of transient expression windows, bypass of feedback repression, supraphysiologic dosage effects, altered TF stoichiometry, and context-dependent engagement of regulatory circuits. These modes provide a rationale for using GOF perturbation to identify regulatory activities that are constrained or incompletely realized under physiological conditions. However, unbiased, single-cell-resolved interrogation of GOF perturbations in antigen-specific T cells *in vivo* has remained limited, due in part to the immunogenicity of transduced donor cells in syngeneic hosts and technical challenges in resolving perturbation effects at cellular resolution^30-32^. We therefore reasoned that systematic GOF perturbation of TFs in antigen-specific CD8^+^ T cells could uncover regulatory activities not readily inferred from expression patterns or LOF genetics.

Guided by this rationale, we developed a GOF screening framework to systematically overexpress TFs in CD8^+^ T cells, independent of endogenous chromatin accessibility and without requiring immunogenic CRISPRa-based systems. Building on prior *in vitro* GOF approaches^20^, we engineered single-cell GOF sequencing (scGOF-seq), a platform for multiplexed *in vivo* profiling of TF GOF perturbations in antigen-specific CD8^+^ T cells across immunocompetent models of infection and cancer. scGOF-seq enabled single-cell-resolved comparison of enforced TF activity within shared biological contexts. To support this approach, we developed a non-immunogenic, MSCV-based retroviral open reading frame (ORF) library that achieves efficient T cell transduction, stable *in vivo* expression, preservation of library complexity, and robust assignment of individual perturbations. Applying scGOF-seq to ORF libraries spanning TFs across diverse regulatory contexts—including dynamically regulated TFs, transient regulators, dosage-sensitive factors, and silenced TFs acting as hidden drivers—we uncovered regulatory activities not readily inferred from endogenous expression patterns. These included unexpected roles for developmental regulators typically silenced in T cells such as SOX2, OCT4, NANOG, and GATA2 in shaping CD8^+^ T cell differentiation *in vivo*.

In particular, scGOF-seq revealed an unexpected role for cMyc, a temporally restricted regulator classically associated with T cell activation, metabolism, and early effector commitment^33-36^. Although cMyc expression is rapidly extinguished during CD8^+^ T cell differentiation^37^, sustained cMyc GOF outside this native temporal window generated a stem-like, effector-featured state with reduced terminal exhaustion, enhanced metabolic fitness and profound antigen-dependent expansion. Building on these findings, we leveraged scGOF-seq to identify cooperating TF modules, including BATF, that complemented cMyc-driven programs and improved T cell responses in solid tumors. Collectively, these results establish scGOF-seq as a framework for uncovering latent and temporally constrained regulatory activities that can be harnessed for rational T cell reprogramming.

## scGOF-seq maps GOF perturbations in CD8^+^ T cells *in vivo*.

Despite advances in multiplexed GOF screening technologies^20-27,38,39^, systematic interrogation of GOF phenotypes in immunocompetent *in vivo* settings remains limited. This gap is important because GOF perturbations can do more than reinforce endogenous T cell programs, such as the stemness-associated activities of TCF1 and FOXO1^40-47^; they can also reveal regulatory activities that are inaccessible under physiological expression patterns, endogenous feedback, or LOF genetics. Defining these activities requires approaches that link enforced gene expression to CD8^+^ T cell state at single-cell resolution *in vivo*.

**Extended Data Fig. 1 ∣:**
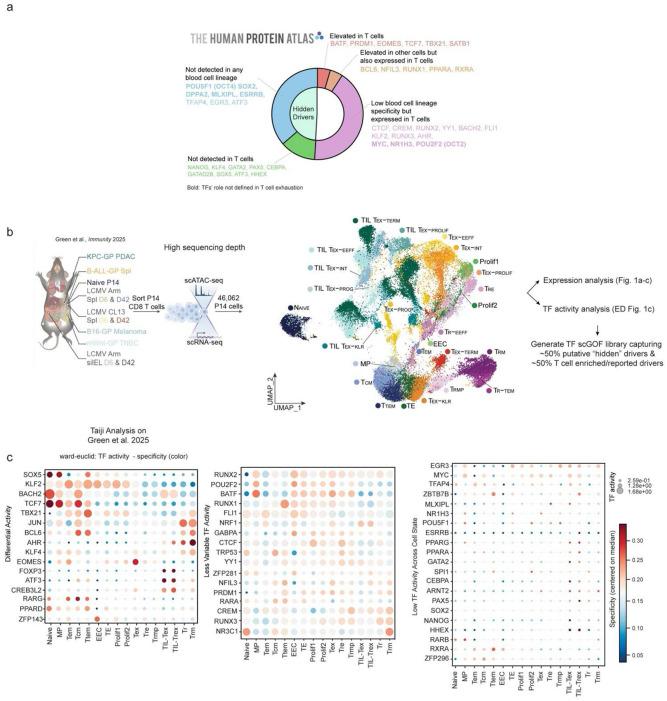
Expression patterns and predicted activity of TFs investigated in scGOF-seq screens. **a,** Classification of TFs based on expression abundance in human T cells using data from The Human Protein Atlas. TFs were grouped as enriched in T cells, expressed but not enriched, low blood-lineage specificity but detectable in T cells, or undetectable in T cells. TFs without previously defined roles in T cell exhaustion are highlighted. **b,** Integrated single-cell transcriptomic and chromatin accessibility datasets from infection and multiple tumor models were used to define CD8^+^ T cell TF activity landscapes. **c,** Bubble plots showing predicted TF activity across CD8^+^ T cell states. Representative TFs are separated by differential (left), low-variance (middle), or uniformly low activity patterns (right).

**Extended Data Fig. 2 ∣:**
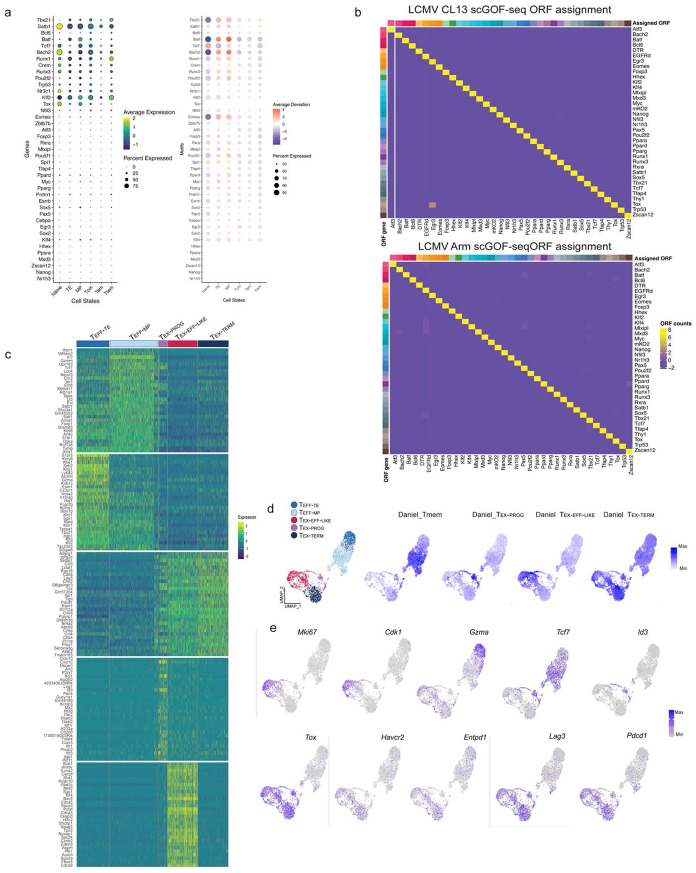
scGOF-seq performance and TF activity landscapes in CD8^+^ T cells during LCMV infection. **a,** Gene expression analysis across CD8^+^ T cell states in Arm infection for selected TFs included in the scGOF-seq library (left). Chromatin accessibility deviation scores (ChromVAR) and percent expression of corresponding target genes are shown on the right. **b,** ORF assignment matrix from scGOF-seq demonstrating expression values of each ORF in assigned cells from Arm and CL13 infection models. **c,** Gene module enrichment analysis for effector exhausted (Tex-eff), progenitor exhausted (Tex-prog), intermediate exhausted (Tex-prog), and terminal exhausted (Tex-term) CD8^+^ T cell states in scGOF-seq screens shown in Fig. 1d. **d-e,** UMAP space representation, gene signature enrichment (e) of feature plots of key marker genes from Fig. 1d.

**Fig. 1 ∣:**
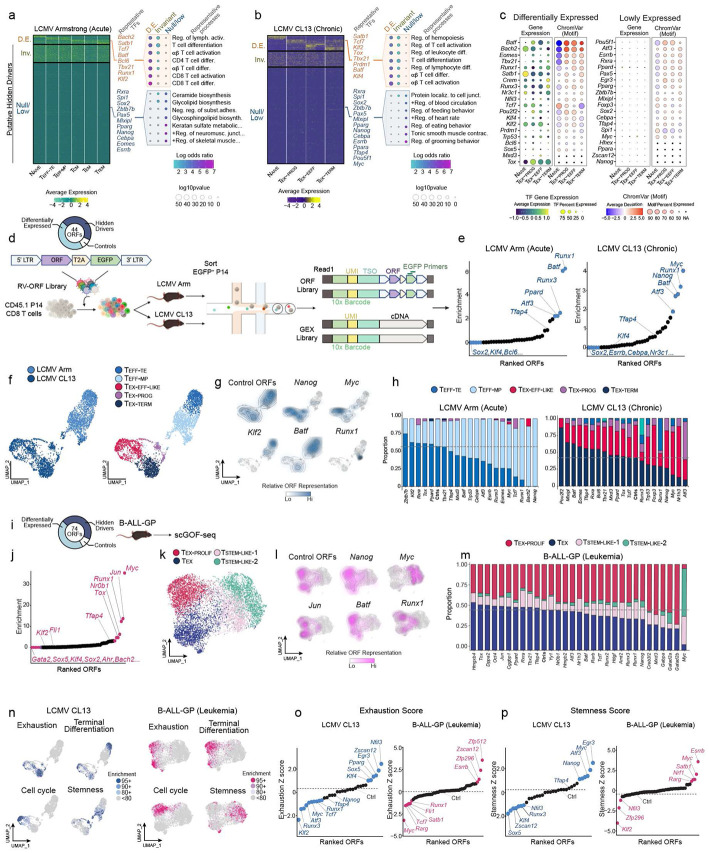
scGOF-seq maps GOF perturbations in CD8^+^ T cells *in vivo*. **a,** Expression dynamics of differentially expressed (DE), invariant (Inv.) and lowly expressed genes across canonical CD8^+^ T cell states during LCMV Armstrong (Arm) infection based on reanalysis of GSE290082, with representative transcription factors (TFs) highlighted (left). Representative biological processes enriched among DE and lowly expressed genes identified by gene ontology analysis are shown (right). **b,** Expression dynamics of DE, Inv. and lowly expressed genes across canonical CD8^+^ T cell states during LCMV Clone 13 (CL13) infection based on reanalysis of GSE290082, with representative TFs highlighted (left). Representative enriched biological processes identified by gene ontology analysis are shown (right). **c,** Gene expression and motif deviation scores derived from paired scRNA-seq and scATAC-seq profiling (GSE290082) for TFs included in the initial screening library. TFs lacking annotated motifs are indicated as NA. **d,** Experimental schematic of scGOF-seq screening. P14 CD8^+^ T cells were transduced with a retroviral ORF library and transferred into mice infected with Arm or CL13. EGFP^+^ donor cells were isolated 2 weeks post-infection for 5′ scRNA-seq analysis. ORF identities were assigned through amplification of EGFP-containing transcripts. **e,** Relative representation of individual ORFs in Arm and CL13 screens normalized to input frequencies. **f,** UMAP representation of scGOF-seq data colored by condition and annotated T cell states. **g,** Representative projection of ORF abundance onto the UMAP shown in (f). **h,** Cell-state composition of selected ORFs with sufficient cellular recovery. **i,** Experimental schematic of expanded scGOF-seq screen (74 vectors) in the B-ALL-GP model 10 d after T cell transfer. **j,** Relative representation of each ORF in the B-ALL-GP screen normalized to input frequencies. **k,** UMAP representation of B-ALL-GP scGOF-seq data colored by condition and annotated T cell states. **l,** Representative projection of ORF abundance onto the UMAP shown in (k). **m,** Cell-state composition for ORFs with sufficient cellular recovery from the screen shown in (i). **n,** Gene-module enrichment scores for exhaustion, stemness, cell cycle and terminal differentiation programs in CL13 (left) and B-ALL-GP (right). **o,** Gene-module enrichment scores for exhaustion signatures for individual ORFs in CL13 screen (left) and B-ALL-GP screen (right). **p,** Gene-module enrichment scores for stemness signatures for individual ORFs in CL13 screen (left) and B-ALL-GP screen (right). For scGOF-seq screens, donor cells were pooled from n = 5-11 mice in (d) and n = 2 mice in (i).

TFs are uniquely suited for probing this unmapped regulatory landscape, as ectopic expression can bypass endogenous epigenetic boundaries to engage alternative gene-regulatory networks. To systematically nominate candidate TFs with latent regulatory capacity, we integrated protein-coding expression data from the Human Protein Atlas, bulk RNA-seq, ImmGen, and high-coverage single-cell RNA-seq datasets (~80,000 reads per cell) spanning murine infection and cancer models^48-50^. This analysis revealed that a substantial fraction of annotated TFs are expressed at trace or relatively low levels in CD8^+^ T cells (Fig. 1a,b; Extended Data Fig. 1a,b). Many of these lowly expressed TFs are linked to non-immune lineage specification or cellular processes not associated with T cell biology, highlighting their potential to engage alternative gene-regulatory programs when overexpressed. Notably, many retain accessible DNA-binding motifs across CD8^+^ T cell chromatin landscapes (Fig. 1c; Extended Data Fig. 1c, 2a), raising the possibility that enforced expression could engage dormant regulatory circuitry. These observations guided selection of TFs spanning complementary regulatory classes: differentially expressed T cell regulators, lineage-silenced developmental TFs with potential latent activity^51^, and temporally restricted TFs whose enforced expression could extend activity beyond their native expression windows (Fig. 1c).

To systematically interrogate GOF perturbations *in vivo*, we developed single-cell GOF sequencing (scGOF-seq), a pooled screening framework that links enforced ORF expression to transcriptional state at single-cell resolution in immunocompetent hosts. We generated a retroviral ORF-T2A-EGFP library of ~45 vectors, including four controls, spanning differentially expressed T cell regulators, lineage-silenced developmental TFs, and temporally restricted TFs (Fig. 1d; Extended Data Fig. 2a). Retrovirus was produced in an arrayed format and used to transduce T cell receptor (TCR) transgenic P14 CD8^+^ T cells specific for the LCMV GP_33-41_ epitope^52^. Transduced cells were quantified for input representation, pooled, and adoptively transferred into recipient mice infected with either acutely resolving LCMV Armstrong (Arm) or persistent LCMV Clone 13 (CL13), established models for probing T cell differentiation (Fig. 1d). Two weeks post-infection, transduced (EGFP^+^) P14 cells were isolated from tissues and subjected to scRNA-seq, enabling assignment of ORF identity at single-cell resolution. As an initial validation, scGOF-seq recapitulated established regulatory logic of CD8^+^ T cell differentiation *in vivo*. GOF of BATF, RUNX1, and AP-4 (encoded by *Tfap4*) increased representation across both acute and chronic infection, whereas KLF2 GOF was selectively depleted under chronic antigen exposure, consistent with prior reports^50,53^ (Fig. 1e).

scGOF-seq allowed transcriptional state investigation of each cell (Fig. 1f; Extended Data Fig. 2b). Unsupervised clustering and gene-signature enrichment analysis^3,54^ resolved major CD8^+^ T cell states, including terminal effector cells (Teff-te), memory precursor effector T cells (Teff-mp), terminally exhausted cells (Tex-term), progenitor exhausted cells (T_ex-prog_), and effector-like exhausted cells (Tex-eff-like)^50^ (Fig. 1f; Extended Data Fig. 2c-e). State-resolved analysis further confirmed known associations, including enrichment of GOF cells with RUNX1 and RUNX3 in memory precursor (Teff-mp) populations, T-bet (encoded by *Tbx21*) and KLF2 in terminal effector (Teff-te) cells, and BATF in early terminally exhausted (Tex-term) cells^9,12,55^ (Fig. 1g,h). These results establish that scGOF-seq captures known CD8^+^ T cell regulatory logic *in vivo* while enabling direct comparison of enforced TF activities within shared biological contexts.

Apart from these anticipated patterns, scGOF-seq uncovered unforeseen *in vivo* activities in TFs that had no previously predicted function in CD8^+^ T cells. ATF3 GOF increased P14 representation in both acute and chronic infection, whereas GOF of NANOG, a pluripotency-associated TF transcriptionally silenced in CD8^+^ T cells, selectively enhanced P14 accumulation during chronic infection (Fig. 1e). cMyc, a transient activation-associated TF with low expression in exhausted T cells (Fig. 1c), preferentially enhanced accumulation in CL13 relative to Arm. These results identify lineage-silenced and temporally restricted TFs whose enforced expression alters antigen-specific T cell accumulation *in vivo*.

**Extended Data Fig. 3 ∣:**
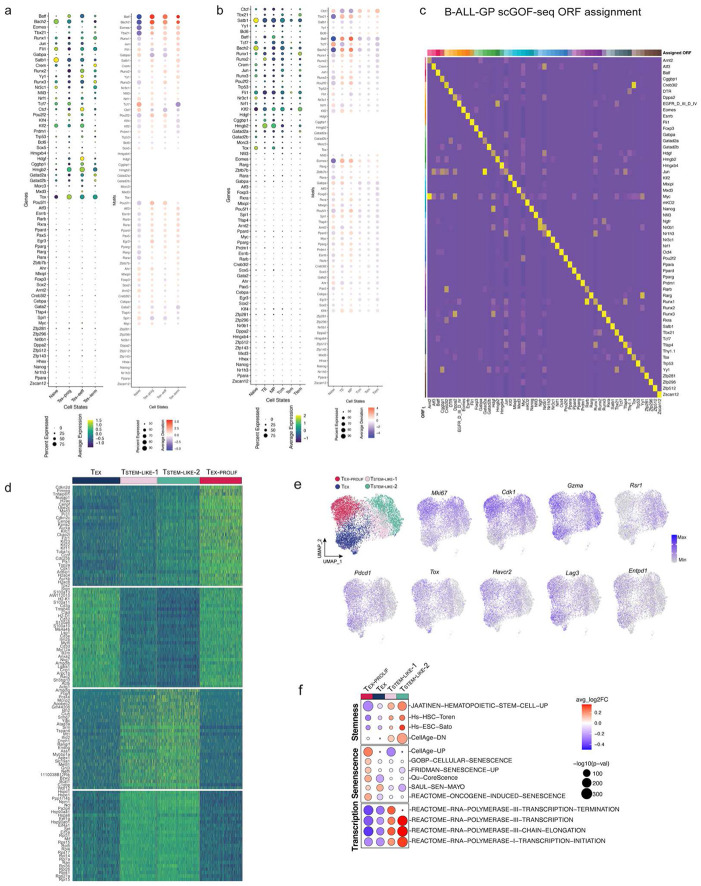
Supplemental scGOF-seq data in B-ALL-GP. **a,b,** Expression and motif accessibility profiles of TFs included in the expanded scGOF-seq library in CL13 (a) and Arm infection (b). **c,** ORF assignment matrix from the B-ALL-GP scGOF-seq. **d,** Gene module enrichment analysis for Tex-term, Tstem-like 1, Tstem-like 2, and Tex-prolif CD8^+^ T cell states from B-ALL-GP33 scGOF-seq. **e,** Marker gene expression. **f,** Pathway enrichment for each subset identified in B-ALL-GP scGOF-seq data.

Having established that scGOF-seq could resolve TF-dependent effects on CD8^+^ T cell differentiation during viral infection, we next asked whether this approach could identify regulatory programs shaping T cell responses in cancer. We therefore applied scGOF-seq to a syngeneic BCR-ABL *Arf*^−/−^ B cell acute lymphoblastic leukemia model expressing GP_33–41_ (B-ALL-GP)^50^ (Fig. 1i) and expanded the screening library to ~75 ORFs (Fig. 1i; Extended Data Fig. 3a-c). Established regulators including JUN, TOX, AP-4, and RUNX1 enhanced T cell accumulation, as expected^28,34,53,56^ (Fig. 1j). Single-cell analysis resolved exhausted (Tex), proliferative exhausted (Tex-prolif), and two stem-like (Tstem-like) populations (Fig. 1k, Extended Data Fig. 3d-f), permitting analysis of how each TF modulated exhaustion specification (Fig. 1l,m). Strikingly, cMyc GOF drove a ~35-fold expansion of tumor-specific CD8^+^ T cells (Fig. 1j). Unbiased scGOF-seq profiling further revealed that cMyc GOF induced a distinct T_stem-like_ 2 population in the B-ALL-GP model characterized by reduced exhaustion and terminal differentiation alongside increased enrichment of stemness features and transcriptional capacity (Fig. 1k-n; Extended Data Fig. 3f). Notably, these state-specific effects would be largely obscured in bulk screens, highlighting the ability of scGOF-seq to uncover unexpected or suppressed regulators of T cell fate.

**Extended Data Fig. 4 ∣:**
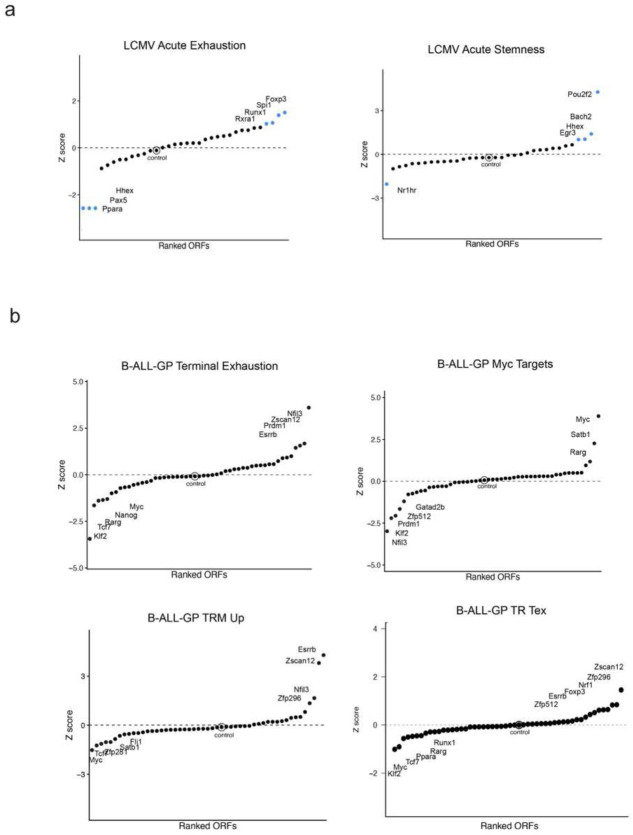
Signature analysis of scGOF-seq. **a,** Ranked ORF enrichment from Arm scGOF-seq based on exhaustion and stemness gene signatures. **b,** Ranked ORF enrichment from B-ALL-GP scGOF-seq across terminal, tissue residency, tumor-resident exhausted and cMyc-target gene signatures.

Across infection and leukemia models, state-resolved analyses further showed that GOF perturbations reshaped the balance between stemness and terminal exhaustion in distinct ways (Fig. 1n-p, Extended Data Fig. 4a,b). As expected, SATB1 and TCF1 (encoded by *Tcf7*) GOF promoted stem-like programs and reduced exhaustion-associated states, consistent with their known roles^40,44-47,57,58^ (Fig. 1o,p). In addition, scGOF-seq uncovered previously unappreciated regulators of exhaustion differentiation. ATF3 GOF reduced exhaustion-associated states and promoted stemness-associated features in LCMV CL13, whereas ZSCAN12 GOF induced exhaustion programs across both LCMV CL13 and B-ALL-GP. NANOG, a lineage-silenced developmental regulator (Fig. 1c), selectively enhanced stemness-associated programs and reduced exhaustion during chronic infection (Fig. 1o,p). Further, cMyc, a temporally restricted regulator, produced similar stemness-enhancing and exhaustion-limiting effects across both chronic infection and leukemia (Fig. 1o,p). Together, these findings show that scGOF-seq captures known regulators of CD8^+^ T cell differentiation while uncovering lineage-silenced and temporally restricted TF activities that reshape exhaustion and stemness programs *in vivo*.

## scGOF-seq uncovers novel GOF perturbations that regulate exhaustion differentiation *in vivo*.

**Fig. 2 ∣:**
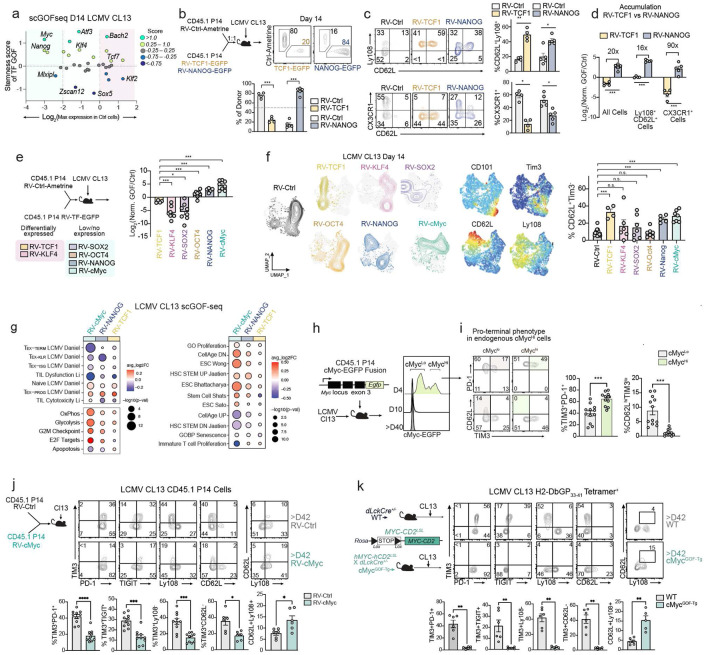
scGOF-seq uncovers latent programs that shape exhaustion differentiation *in vivo*. **a,** Computed stemness score for each TF from the CL13 scGOF-seq screen plotted by expression level of each TF in Ctrl cells. Genes with low or trace expression are marked by grey or blue shaded regions. **b,** RV-control, RV-TCF1, or RV-NANOG P14 donor cells were co-transferred into CL13-infected mice. Donor cell frequency was assessed in spleens at day 14 of infection. **c,** Representative flow cytometry plots of donor cells from (b). **d,** Normalized accumulation of RV-TCF1 versus RV-NANOG cells (both normalized to RV-Ctrl cells). **e,** In a mixed transfer approach, RV-TCF1, RV-KLF4, RV-SOX2, RV-OCT4, RV-NANOG, RV-cMyc cells were co-transferred with RV-Ctrl cells into CL13-infected mice (left). Accumulation of donor cells relative to RV-Ctrl cells were assessed on day 14 of CL13 infection (right). **f,** High-dimensional spectral flow cytometry UMAP representation and clustering of donor cells (left) with percentage of CD62L^+^Tim3^−^ stem-like cells quantified (right) from (e). **g,** Comparison of gene stemness, cell cycle, and metabolic associated programs between RV-cMyc, RV-NANOG, and RV-TCF1 cells recovered from the CL13 scGOF-seq screen. **h,** cMyc-eGFP fusion P14 cells were transferred to mice infected with CL13. cMyc-EGFP levels were assessed in donor cells on days 4, 10, and >40 post-infection. **i,** Flow cytometry phenotype of cMyc-EGFP^Hi^ and cMyc-EGFP^Lo^ cells on day 4 of CL13 infection. **j,** RV-Ctrl and RV-cMyc P14 cells were mixed and transferred into CL13-infected mice. Donor cells were profiled >42 days post-infection via spectral flow cytometry. **k,** Littermate WT and cMyc^GOF-Tg^ transgenic mice were infected with CL13. Tetramer-positive cells were analyzed by spectral flow cytometry >42 d post-infection. Graphs show mean ± SEM of n=4-12 mice from one representative experiment or pooled from two or more intendent experiments (b-k). *p<0.05, **p<0.005, ***p<0.001, unpaired (d,e,f,h,k) and paired Student’s t-test (b,c,i,j).

Although scGOF-seq uncovered broad GOF activity across the TF repertoire, the principles organizing these activities remained unclear. We reasoned that TF GOF effects could arise through at least two distinct mechanisms: amplification of endogenous regulatory programs already used during T cell differentiation, or activation of latent regulatory activities that are normally inaccessible, silenced, or temporally constrained in T cells. To distinguish these possibilities, we compared endogenous TF expression with GOF potency for promoting stemness-associated phenotypes during chronic infection (Fig. 2a). TFs with established roles in T cells, including BACH2, TCF1, and KLF2, displayed expected stemness-promoting activity, consistent with reinforcement of native differentiation programs^40,45-47,58-61^. In contrast, several TFs with low or undetectable expression in exhausted CD8^+^ T cells, including NANOG and ATF3, emerged as potent regulators when ectopically expressed. cMyc also displayed strong GOF potency despite low expression at day 14 of LCMV CL13 infection, suggesting that some latent regulatory activities may not be lineage-silent but instead constrained to specific temporal windows during physiological differentiation. Thus, discordance between endogenous expression and GOF activity revealed a hidden regulatory landscape that is not readily inferred from native T cell trajectories alone.

**Extended Data Fig. 5 ∣:**
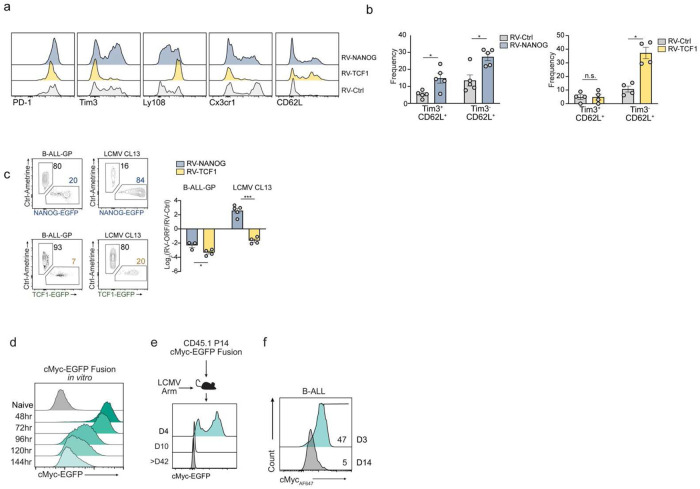
Phenotype of NANOG GOF, TCF1 GOF, and cMyc expression kinetics. RV-NANOG, RV-TCF1, or RV-Ctrl P14 were mixed 1:1 and transferred into mice infected with CL13 or mice bearing ALL-GP tumors. **a-b**, Histograms comparing PD-1, Tim3, Ly108, Cx3cr1, and CD62L expression between donor cells on day 14 of CL13. Frequency of Tim3^+^CD62L^+^ and Tim3^−^CD62L^+^ cells between RV-ctrl and RV-Nanog (left), and RV-ctrl and RV-TCF1 (right) donor cells isolated from LCMV CL13 mice. Accumulation of RV-ctrl, RV-TCF1, and RV-NANOG p14 cells isolated day 10 post transfer from B-ALL-GP bearing mice (left) or at day 14 post transfer from CL13-infected mice (c). **d,** cMyc–EGFP fusion CD8 T cells were stimulated in vitro and expression kinetics of cMyc-EGFP were assessed by spectral flow cytometry. **e**, cMyc-eGFP fusion P14 cells were transferred to mice infected with Arm. cMyc-EGFP levels were assessed in donor cells on days 4, 10, and >40 post-infection. **f,** cMyc expression dynamics in CD8+ T cells in the B-ALL model assessed by intracellular cMyc antibody staining at day 3 and day 14 post infection. Graphs show mean ± SEM of n=4-5 mice from one representative experiment or pooled from two or more intendent experiments. *p<0.05, ***p<0.001, unpaired or paired Student’s t-test.

Building on this distinction, we focused first on NANOG as a prototypical lineage-latent regulator. NANOG was particularly striking because it was transcriptionally silent in CD8^+^ T cells (Fig. 1a-c), yet selectively enhanced stemness-associated phenotypes and accumulation during chronic infection (Fig. 1e,p). To determine whether this activity simply mimicked a canonical T cell stemness program or instead reflected a distinct latent regulatory output, we compared NANOG GOF with TCF1 GOF, a canonical endogenous driver of T cell stemness and progenitor-like exhausted T cells^40,58^ (Fig. 2b). In LCMV CL13 and B-ALL-GP, both NANOG and TCF1 increased progenitor-like populations including CD62L^+^Ly108^+^ cells, which have been reported to exhibit robust stem-like CD8^+^ T cell potential^62^ (Fig. 2c, top; Extended Data Fig. 5a,b). However, their functional outputs diverged. TCF1 GOF reinforced a TEX-PROG-like state and limited accumulation of CX3CR1^+^ intermediate exhausted cells that retain effector function (TEX-INT)^2,63,64^, whereas NANOG GOF combined stemness-associated programming with substantial accumulation during chronic infection (Fig. 2c,d). While TCF1 is a potent driver of canonical stem-like differentiation^40,58^ NANOG GOF produced a 16-fold increase in Ly108^+^CD62L^+^ stem-like cells together with a 90-fold expansion of CX3CR1^+^CD62L^+^ Tex-int cells relative to TCF1 GOF (Fig. 2d). Consistent with our scGOF-seq findings (Fig. 1e,j), NANOG GOF-induced accumulation was context dependent, occurring in CL13 but not B-ALL-GP (Extended Data Fig. 5c). Thus, NANOG GOF reveals a latent regulatory program that supports progenitor-like features without restricting downstream Tex-int accumulation, generating differentiation outputs not achieved by simply amplifying a canonical endogenous stemness program.

Because NANOG belongs to a broader network of pluripotency-associated TFs, we next asked whether similar activity extended to other Yamanaka-associated factors, including SOX2, OCT4, KLF4, and cMyc (also included in our scGOF-seq screens, (Fig. 1d,i)). Comparative analysis in chronic infection revealed divergent effects despite their shared developmental associations (Fig. 2e). SOX2 and KLF4 GOF broadly impaired T cell accumulation, OCT4 GOF induced a 3-fold accumulation, whereas NANOG and cMyc promoted robust accumulation (7-fold and 35-fold relative to control cells, respectively) under chronic antigen exposure. NANOG and cMyc also limited terminal exhaustion while enhancing stemness-associated features (Fig. 2f). Notably, this latent regulatory output could not be predicted from developmental identity alone as related TFs encoded distinct, and in some cases opposing, effects on T cell differentiation and accumulation.

Although cMyc, NANOG, and TCF1 each induced stemness-associated programs (Fig. 2f), cMyc produced a broader transcriptional response that coupled progenitor-like features with reduced terminal exhaustion and enhanced fitness-associated pathways (Fig. 2g). Specifically, cMyc GOF decreased Tex-term signatures while increasing programs associated with oxidative phosphorylation, glycolysis, proliferation, embryonic stem cell identity, and survival. Thus, unlike TCF1 that primarily reinforced stemness-associated states, cMyc GOF integrated stem-like differentiation with suppression of terminal exhaustion and induction of metabolic and proliferative programs.

These findings identified cMyc as a temporally constrained mode of hidden regulatory activity. Unlike lineage-silent TFs such as NANOG, cMyc is normally transient and rapidly extinguished after T cell activation, as we demonstrated *in vitro* and *in vivo* in CL13, Arm, and B-ALL-GP models (Fig. 2h; Extended Data Fig. 5d-f). This is consistent with tight temporal regulation of its physiological activity^37,65,66^. In agreement with prior studies, cells with high endogenous cMyc preferentially adopted terminally differentiated or exhausted phenotypes, indicating that transient endogenous cMyc activity is linked to effector commitment under physiological conditions (Fig. 2i). In contrast, sustained cMyc GOF outside its native temporal window promoted robust stem-like features during chronic infection (Fig. 2j). Direct comparison of endogenous and sustained cMyc activity therefore revealed opposing phenotypes where transient endogenous cMyc expression was associated with more terminal differentiation (in line with sort transfer experiments^35,67^), whereas artificially sustained expression promoted stemness and restrained terminal exhaustion.

**Extended Data Fig. 6 ∣:**
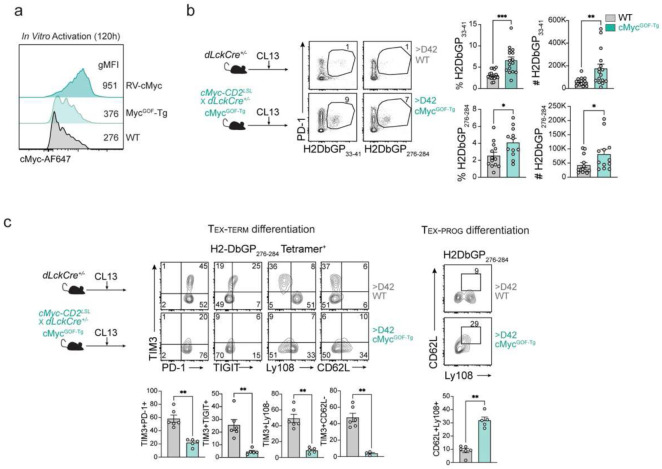
cMyc GOF limits terminal exhaustion in tetramer^+^ CD8^+^ T cells. **a,** cMyc expression kinetics following *in vitro* activation of RV-cMyc CD8^+^ T cells or CD8^+^ T cells isolated from Myc^GOF-Tg^ and wild-type mice. **b,** Wild-type and Myc^GOF-Tg^ mice were infected with CL13 and tetramer^+^ CD8^+^ T cells were analyzed by spectral flow cytometry at >42 days post-infection. Enumeration of total H2DbGP_33-41_ and H2DbGP_276–284_ tetramer^+^ cells from Wild-type and MycGOF-Tg mice. **c,** Representative flow cytometry plots and phenotype of H2DbGP_276–284_ tetramer^+^ cells from (b). Graphs show mean ± SEM of n=3-16 mice from one representative experiment or pooled from two or more independent experiments. *p<0.05, **p<0.005, ***p<0.001, unpaired or paired Student’s t-test.

To test whether sustained cMyc also reshaped endogenous antiviral T cell responses in addition to transgenic P14 cells, we generated cMyc^GOF-Tg^ mice by crossing cMYC-CD2-LSL mice with distal Lck-Cre mice (Fig. 2k). Despite lower cMyc expression than retroviral-mediated overexpression (Extended Data Fig. 6a), cMyc^GOF-Tg^ mice showed increased accumulation of GP33^+^ and GP276^+^ CD8^+^ T cells during chronic infection, with markedly reduced exhaustion phenotypes and increased Ly108^+^CD62L^+^ TEX-PROG populations (Fig. 2k, Extended Data Fig. 6b,c). Thus, sustained cMyc activity restrains terminal exhaustion and preserves stem-like differentiation in both adoptively transferred and endogenous CD8^+^ T cells.

Together, these findings reveal emerging principles of latent regulatory activity in CD8^+^ T cells. First, stemness-associated programs can be induced through distinct regulatory routes, as NANOG, cMyc, and TCF1 produced overlapping but non-identical effects on progenitor-like phenotypes, differentiation, and accumulation. Second, developmental identity alone did not predict GOF activity, as related pluripotency-associated TFs exerted divergent and sometimes opposing effects on T cell fitness. For example, KLF4 and SOX2 blunted exhausted cell accumulation, whereas OCT4, NANOG, and cMyc enhanced accumulation in CL13. Third, scGOF-seq highlighted several modes of regulatory potential exposed through enforced TF activity, including lineage-latent activities encoded by TFs normally silenced in T cells and temporally constrained activities exemplified by cMyc, whose sustained expression outside its native window coupled stem-like differentiation with suppression of terminal exhaustion and activation of metabolic and proliferative programs. Thus, physiological T cell differentiation masks a broader regulatory landscape with therapeutic potential that can be uncovered by systematic GOF perturbation.

## Sustained cMyc expression diverts exhausted T cells into a stem-like effector trajectory.

**Extended Data Fig. 7 ∣:**
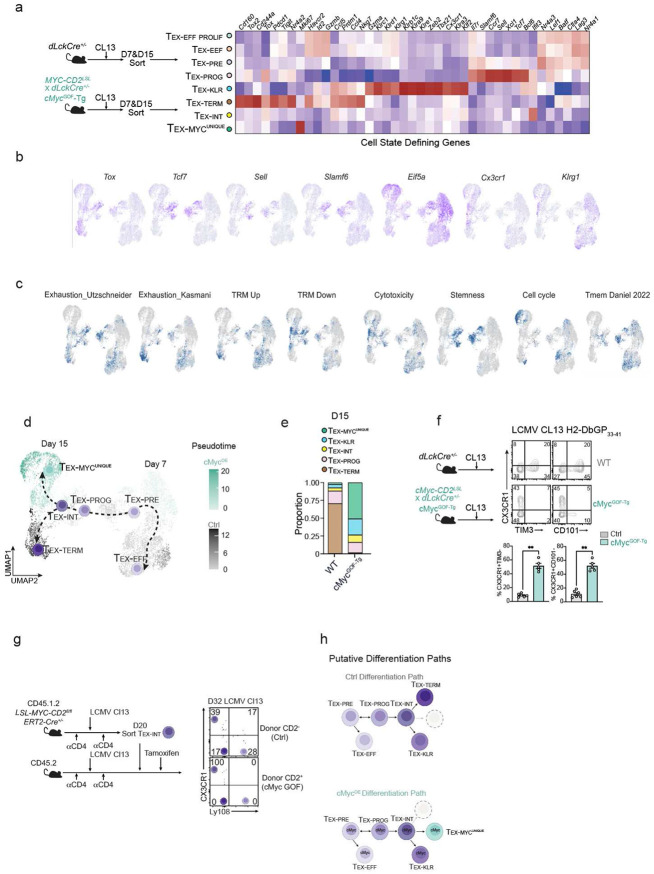
cMyc GOF induces an alternative exhausted-linage differentiation trajectory. **a,** Expression of representative cell state-defining genes used to annotate cells in scRNA-seq data from Fig. 3b. **b,** Feature plots showing expression of marker genes defining CD8^+^ T cell differentiation states from Fig. 3b. **c,** Feature plots displaying gene-signature scores across CD8^+^ T cell populations from Fig. 3b. **d,** UMAP projections illustrating the emergence of unique cells induced by cMyc GOF during CL13 and divergence of differentiation trajectories between wild-type and cMyc GOF CD8^+^ T cells. **e**, Relative proportion of each cell annotate state on day 15 of CL13 infection from (Fig. 3b). **f,** CX3CR1 phenotype of tetramer^+^ CD8 T cells in WT and cMyc^GOF-Tg^ mice during CL13. **g,** Tex-int cells (PD-1+CX3CR1+Ly108−KLRG1−) were sorted from WT or inducible-cMyc^GOF-Tg^ mice (CD45.1/CD45.2 LSL-MYC-CD2; Cre-ERT2) and transferred into infection matched recipients with CD4 depletion to ensure deep exhaustion. Following transfer, mice were treated with tamoxifen and then 10 days following tamoxifen treatment, cMyc GOF and Ctrl cells within the same recipient mouse were analyzed by spectral flow cytometry. **h,** Inferred differentiation trajectories based on cMyc^GOF-Tg^ scRNA-seq profiling data. Graphs show mean ± SEM of n=5-6 mice from one representative experiment or pooled from two or more independent experiments. **p<0.005, unpaired Student’s t-test.

**Fig. 3 ∣:**
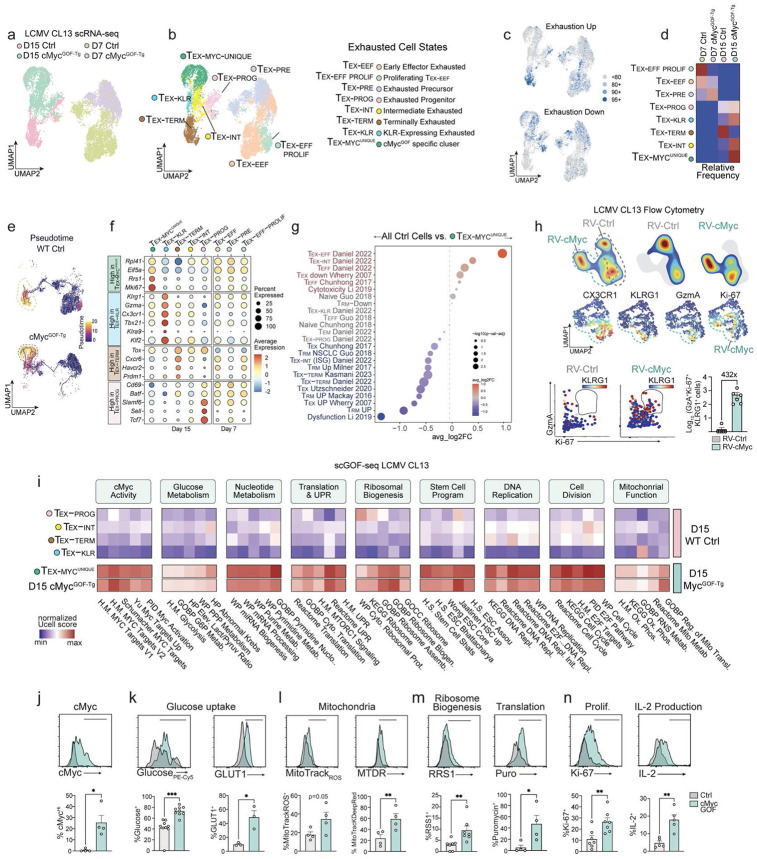
cMyc GOF diverts exhausted T cells into a stem-like effector trajectory. **a,** WT and cMyc^GOF-Tg^ mice were infected with CL13, and CD8^+^ T cells were sort-purified for scRNA-seq analysis on days 7 and 15 of infection. UMAP representation and clustering of CD8^+^ T cells by condition. **b,** UMAP representation and annotation of cell states from (a). **c,** Enrichment of genesets elevated in exhausted T cells (exhaustion up) and downregulated in exhausted cells relative to functional effector cells (exhaustion down). **d,** Relative abundance of each cell state. **e,** Trajectory and pseudotime analysis of WT cells (top) or cMyc^GOF-Tg^ cells (bottom). Dashed regions indicate distinct terminal differentiation endpoints. **f,** Expression analysis of representative marker genes from annotated cell states from (a). **g,** Gene-set comparison between WT control cells and the Tex-myc^UNIQUE^ population from (a). **h,** RV-cMyc and RV-Ctrl P14 cells were mixed and transferred into congenic mice infected CL13. Splenocytes were profiled on days 14-16 of infection via spectral flow cytometry. UMAP representation and clustering of RV-Ctrl and RV-cMyc cells (top) and expression levels of GzmA, KLRG1, CX3CR1 and Ki-67 are indicated (middle). Quantification of GzmA^+^Ki-67^+^KLRG1^+^ donor cells (bottom). **i,** Pathway-enrichment analysis comparison of WT states, cMyc^GOF-Tg^ cells, and Tex-myc^UNIQUE^ cells from (a). **j–n,** Representative histograms and quantitative analyses of cMyc expression, glucose uptake, GLUT1 expression, mitochondrial ROS, mitochondrial mass (MitoTracker Deep Red), RRS1 expression, puromycin incorporation, Ki-67 expression, and IL-2 production in RV-cMyc versus RV-Ctrl donor cells during CL13 infection on days 12-16 post-infection. Graphs show mean ± SEM of n=4-9 from one representative experiment or pooled from two or more independent experiments (h,j-n). *p < 0.05, **p < 0.005, ***p < 0.001, paired Student’s t-test.

To resolve how cMyc GOF reshapes exhaustion trajectories, we performed scRNA-seq on polyclonal CD8^+^ T cells isolated from cMyc^GOF-Tg^ and control mice on days 7 and 15 of LCMV CL13 infection (Fig. 3a). Cells segregated by timepoint as expected, and annotation using unbiased clustering and established gene expression signatures identified canonical exhausted states, including Tex-eff, Tex-pre, Tex-prog, Tex-int, Tex-klr, and Tex-term^3^ (Fig. 3b; Extended Data Fig. 7a-b). Consistent with our flow cytometry profiling (Fig. 2 j-k; Extended Data Fig. 7b-c), scRNA-seq analysis indicated that cMyc GOF restrained exhaustion programs (Fig. 3c), markedly reducing formation of TEX-TERM cells, and increasing Tex-prog, Tex-int and Tex-klr states (Fig. 3d). Trajectory analysis revealed a bifurcation at the Tex-int stage: control cells predominantly progressed toward Tex-term, whereas cMyc GOF cells diverted into a distinct branch, termed Tex-myc^Unique^ (Fig. 3e, Extended Data Fig. 7d). Approximately 50% of day 15 cMyc GOF cells occupied this unique state, indicating that sustained cMyc activity diverts a substantial fraction of exhausted CD8^+^ T cells away from the canonical terminal exhaustion trajectory (Extended Data Fig. 7e).

The Tex-myc^Unique^ population shared select effector-associated features with endogenous exhausted effector-like states, including Tex-int, Tex-klr, and Tex-eff, but remained transcriptionally distinct from each of these native populations (Fig. 3b,f,g; Extended Data Fig. 7d,f). Gene-set analysis further showed that TEX-MYC^Unique^ cells were not enriched for canonical Tex-klr or Tex-int signatures, but instead displayed increased proliferation and ribosome- and translation-associated programs, including *Mki67*, *Rpl41*, *Eif5a*, and *Rrs1* (Fig. 3f). Thus, cMyc GOF did not simply expand a conventional Tex-int or Tex-klr population, but generated an alternative effector-like proliferative state with reduced dysfunction-associated programs. Consistent with the transcriptional features of the Tex-myc^Unique^ cluster (Fig. 3f,g; Extended Data Fig. 7b), spectral flow cytometry independently validated this state, showing that cMyc GOF induced a GzmA^+^Ki-67^+^KLRG1^+^ population that was largely absent in controls and present at >400-fold higher frequency (Fig. 3h). Because this population emerged in mixed-transfer settings, its appearance likely reflects cell-intrinsic reprogramming rather than differences in viral burden or tissue environment. Finally, tamoxifen-inducible activation of cMyc GOF in purified Tex-int cells blocked progression toward Tex-term and maintained effector-like cells, whereas control cells followed the expected Tex-int to Tex-term cells^68^ (Extended Data Fig. 7g). Together, these data indicate that sustaining cMyc expression outside its native expression window redirects exhausted CD8^+^ T cells away from terminal exhaustion and toward an effector-like proliferative trajectory (Extended Data Fig. 7h).

## cMyc GOF induces multimodal reprogramming of exhausted CD8^+^ T cells.

cMyc is a canonical regulator of anabolic growth programs^69,70^. In contrast, exhausted CD8^+^ T cells progressively lose proliferative and metabolic capacity during chronic stimulation and experience increasing proteostatic stress^71,72^. Although cMyc is considered a classical regulator of CD8^+^ T cell activation, proliferation, and metabolism in early activated cells^35^, cMyc is rapidly silenced following activation^65,66^ (Fig. 2h; Extended Data Fig. 5d-f). We therefore asked whether sustained cMyc maintains anabolic programs that are normally attenuated during exhaustion.

In WT cells, cMyc-associated pathways were broadly depleted across exhausted state (Fig. 3i). In contrast, cMyc^GOF-Tg^ cells, including Tex-myc^Unique^ cells, maintained enrichment of programs linked to glucose metabolism, mitochondrial function, ribosome biogenesis, translation, proliferation and embryonic stem cells (Fig. 3i). These data suggested that sustained cMyc activity preserves anabolic and fitness-associated programs under chronic antigen stimulation.

We next experimentally validated these transcriptional changes. cMyc GOF cells showed increased glucose uptake and elevated surface GLUT1 expression, indicating enhanced glucose utilization^73^ (Fig. 3j,k). MitoTracker analysis further revealed increased mitochondrial mass and reactive oxygen species production, consistent with augmented mitochondrial activity^74^ (Fig. 3l). Because metabolic fitness is tightly linked to ribosome biogenesis and protein synthesis, we next assessed anabolic output. cMyc GOF cells expressed higher levels of RRS1, a ribosome-biogenesis factor also enriched in the Tex-myc^Unique^ transcriptional program, and showed an approximately 7-fold increase in nascent protein synthesis by puromycin incorporation^75^ (Fig. 3m). Since robust proliferation and effector function depend on extensive remodeling of translational and metabolic programs^76,77^, we assessed whether these changes translated into improved measures of proliferation and fitness. These changes were accompanied by increased Ki-67 expression and enhanced IL-2 production, consistent with elevated proliferative and functional capacity (Fig. 3n).

Notably, cMyc GOF coupled effector-associated molecules, including KLRG1 and GzmA, with elevated glucose uptake, mitochondrial mass and translation. This combination distinguishes cMyc GOF cells from conventional exhausted or senescent-like KLRG1^+^ states^78,79^, in which effector-marker expression is often associated with reduced metabolic fitness. Thus, sustained cMyc activity appears to support an effector-featured state with preserved metabolic capacity during chronic antigen exposure, rather than simply expanding conventional effector-like exhausted populations.

## Stabilized cMyc amplifies antigen-dependent accumulation and metabolic fitness paired with effector function.

**Fig. 4 ∣:**
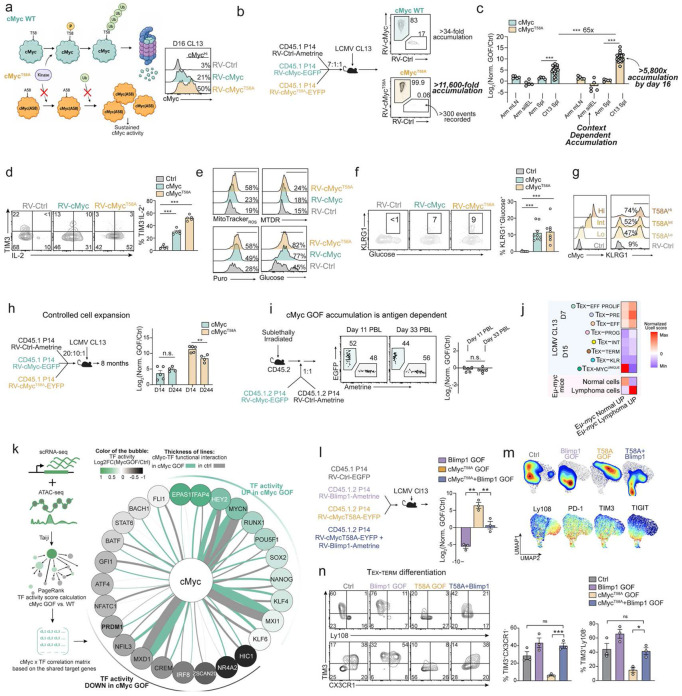
Stabilized cMyc amplifies antigen-dependent accumulation and metabolic fitness paired with effector function. **a,** Model of stabilized cMycT58A. RV-control, RV-cMyc and RV-cMyc^T58A^ P14 cells were co-transferred into mice infected with CL13 and cMyc levels were assessed via flow cytometry on day 16 of infection. **b,c,** Normalized accumulation of RV-cMyc or RV-cMyc^T58A^ cells relative to RV-Ctrl cells in mLN, siIEL, and spleen on days 14-16 of Arm or CL13 infection. **d,** TIM3 expression and IL-2 production during CL13 from (b). **e,** Representative histograms comparing mitochondrial function, glucose uptake, and protein translation in donor cells from b. **f,** Glucose uptake and KLRG1 expression on donor cells from (b). **g,** KLRG1 expression based on graded cMyc expression from (b). **h,** RV-Ctrl, RV-cMyc and RV-cMycT58A P14 cells were co-transferred into mice infected with LCMV CL13. Accumulation of cells was evaluated on days 14 and 244 of CL13 infection. **i,** RV-Ctrl and RV-cMyc donor cells transferred into sub-lethally irradiated hosts. Accumulation of donor cells was assessed on days 11 and 33 post-transfer. **j,** Gene-signature analysis comparing normal lymphocytes from Eμ-myc mice and Eμ-myc-driven malignant lymphoma. Eμ-myc mice express mouse cMyc under the control of the immunoglobulin heavy-chain enhancer Eμ and develop B cell lymphoma. Gene signatures upregulated in non-malignant Eμ-myc lymphocytes or Eμ-myc lymphoma samples were defined from the Sabo et al. dataset^79^, scored in the B-ALL-GP33 scGOF-seq dataset (Fig. 1) and visualized by cell state. **k,** Taiji analysis of computed TF activity in cMyc-GOF cells. Color of circles reflects the predicted activity of each TF in Ctrl cells or cMyc GOF cells (grey/black indicates reduced TF activity in cMyc GOF cells). Line thickness represents the functional interaction or shared target genes between each TF and cMyc in cMyc GOF cells (green) or Ctrl cells (gray). **l,** RV-Ctrl, RV-Blimp1, RVcMyc^T58A^, and RV-Blimp1+cMyc^T58A^ P14 cells were mixed and transferred to mice infected with CL13. Spleens were harvested on days 14-16 post-infection. **m,** Spectral flow cytometry profiling of donor cells from (l). **n,** Representative flow cytometry plots and quantitation of TIM3^+^CX3CR1^−^ and TIM3^+^Ly108^−^ populations. Graphs show mean SEM ± of n=4-20 from one representative experiment or pooled from two or more independent experiments (b-i,l-n). *p < 0.05, **p < 0.005, ***p < 0.001, n.s.=non-significant, paired Student’s t-test.

Having found that sustained cMyc expression redirects exhausted CD8^+^ T cell differentiation, we next asked whether increasing cMyc protein stability would further amplify this program. cMyc abundance is tightly controlled by ubiquitin-mediated degradation, and phosphorylation at T58 promotes rapid turnover (Fig. 4a)^80^. We therefore tested the stabilized cMyc^T58A^ isoform in a triple mixed-transfer experiment with RV-Ctrl, RV-cMyc, and RV-cMyc^T58A^ transduced P14 cells in LCMV CL13-infected mice. As anticipated, cMyc^T58A^ GOF cells exhibited substantially higher cMyc abundance than wild-type cMyc GOF cells *in vivo* (Fig. 4a). We next assessed how this artificially stabilized cMyc activation impacted T cell accumulation in CL13. While wild-type cMyc GOF increased P14 accumulation by 17-fold, cMyc^T58A^ GOF induced a dramatic >5,800-fold expansion of P14 cells, a notable 65-fold increase in the relative accumulation of cMyc^T58A^ cells compared with wild-type cMyc GOF in LCMV CL13 (Fig. 4b). With cMyc^T58A^ GOF, some mice exhibited >11,000-fold enrichment of P14 cells within 16 days of infection, representing an unprecedented degree of accumulation of T cells in the widely used LCMV CL13 model (Fig. 4c).

**Extended Data Fig. 8 ∣:**
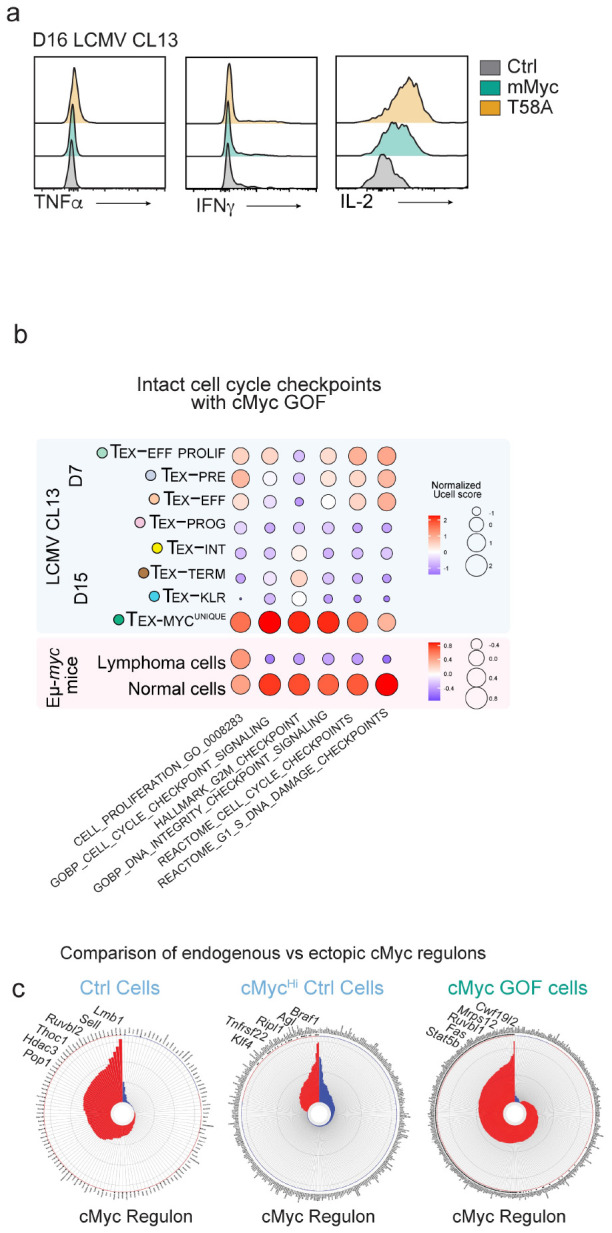
Additional phenotyping of cMyc GOF cells. **a,** Cytokine production (TNFα, IFNγ and IL-2) of RV-ctrl, RV-cMyc, and RV-cMyc^T58A^ cells on day 16 of CL13 infection. **b,** Gene set enrichment scores for cell-cycle checkpoint pathways across exhausted CD8^+^ T cell subsets from scRNA-seq profiling from (Fig. 3a). **c,** scMiner analysis was used to predict context-dependent target genes of cMyc in Ctrl cells, cMycHi (high levels of endogenous *Myc* gene expression), and cMyc^GOF-Tg^ cells from scRNA-seq profiling experiment in (Fig. 3b).

Phenotypically, cMyc^T58A^ GOF broadly reinforced the metabolic and functional programs induced by wild-type cMyc GOF. Compared with cMyc GOF cells, cMyc^T58A^ GOF cells displayed equal or greater mitochondrial mass and activity, glucose uptake, translational output, IL-2 production, reduced inhibitory receptor expression, and increased KLRG1 (Fig. 4d-f), without altering IFNγ or TNFα production (Extended Data Fig. 8a). We also noted a dose-dependent relationship between cMyc abundance and key phenotypes, including KLRG1 expression levels (Fig. 4g). Together, these findings indicate that cMyc abundance quantitatively tunes antigen-dependent accumulation, metabolic fitness, and differentiation during chronic infection.

## cMyc-driven expansion is controlled and antigen-dependent

Despite the oncogenic potential of stabilized cMyc, neither cMyc nor cMyc^T58A^ GOF induced unchecked or antigen-independent CD8^+^ T cell expansion under the conditions tested. Instead, accumulation was strongly context dependent: cMyc and cMyc^T58A^ GOF cells expanded only modestly during acute LCMV Arm infection and did not enrich among donor cells in the small-intestinal epithelium (Fig. 1e, 4c). Long-term follow-up showed no continuous expansion of P14 cells beyond 240 days after transfer (Fig. 4h), and cMyc GOF cells failed to accumulate in irradiated hosts lacking cognate antigen, arguing against antigen-independent homeostatic expansion (Fig. 4i). Moreover, cMyc GOF CD8^+^ T cells did not upregulate cMyc-driven malignant transformation programs^81,82^; rather, these gene sets were reduced relative to controls (Fig. 4j). Cell-cycle checkpoint signatures remained intact, contrasting with cMyc-transformed lymphoma cells, in which these pathways were diminished (Extended Data Fig. 8b). These distinct cMyc-associated functions may, in part, reflect differences between regulons associated with sustained cMyc GOF and endogenous cMyc activity (Extended Data Fig. 8c). Together, these data suggest that stabilized cMyc supports antigen-dependent T cell accumulation and metabolic fitness while remaining constrained by antigen availability, without evidence of autonomous proliferation or overt transformation in the models examined.

## cMyc reprogramming antagonizes Blimp1-dependent exhaustion programs.

cMyc GOF led to multimodal changes in T cell biology, consistent with the broad-acting role of cMyc in transcriptional regulation. To identify regulatory nodes associated with this reprogramming, we integrated ATAC-seq from WT and cMyc^GOF-Tg^ CD8^+^ T cells isolated during LCMV CL13 infection with matched scRNA-seq data and applied Taiji, a framework that infers TF activity from gene-expression and chromatin-accessibility profiles^10,83^ (Fig. 4k). As expected from prior studies linking cMyc to AP-4 induction^34^, Taiji predicted increased AP-4 activity in cMyc GOF cells. In parallel, Blimp1 activity was among the exhaustion-associated programs reduced in the cMyc GOF context. Notably, predicted cMyc and Blimp1 target genes showed increased overlap in cMyc GOF cells compared with controls, suggesting potential antagonism at shared regulatory loci (Fig. 4k).

We prioritized Blimp1 for functional testing because multiple lines of evidence converged on this factor. Blimp1 counteracts cMyc-driven phenotypes in B cells^84-87^, and in CD8^+^ T cells it is a well-established driver of terminal exhaustion that restricts stemness programs^88-91^, represses mitochondrial biogenesis^92^, directly suppresses IL-2 production^93^, and limits proliferation under persistent antigen^94^. These functions are reciprocal to the major phenotypes induced by cMyc GOF. We therefore asked whether restoring Blimp1 activity would be sufficient to oppose cMyc-driven reprogramming. To test this, we performed four-way mixed transfers of P14 cells transduced with RV-Ctrl, RV-Blimp1, RV-cMyc^T58A^, or RV-cMyc^T58A^+RV-Blimp1 into LCMV CL13-infected mice. Concomitant Blimp1 overexpression abrogated key cMyc GOF-induced phenotypes, including enhanced T cell accumulation, stem-like features, effector programs and reduced TIM3 expression (Fig. 4l-n). These data indicate that Blimp1 is sufficient to counteract major components of the cMyc GOF phenotype and support a model in which sustained cMyc activity restrains Blimp1-dependent exhaustion programs while coordinately promoting metabolic, proliferative and differentiation states under persistent antigen stimulation.

## cMyc GOF reprograms T cell differentiation in cancer and enhances leukemia control.

**Fig. 5 ∣:**
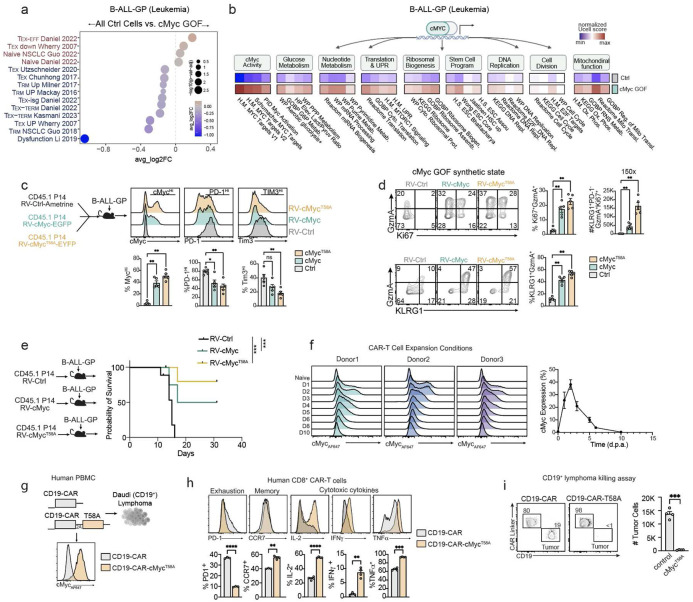
cMyc GOF reprograms anti-tumor responses and enhances cell therapy efficacy in leukemia. **a,** Geneset comparison between Ctrl cells and the cMyc GOF population from B-ALL-GP scGOF-seq. **b,** Pathway-enrichment analysis comparing Ctrl cells and cMyc GOF cells in B-ALL-GP scGOF-seq data. **c,** Expression of cMyc, PD-1 and TIM-3 in RV-control, RV-cMyc and RV-cMyc^T58A^ donor P14 cells transferred into B-ALL-GP-bearing mice and analyzed 10 days after T cell transfer. **d,** Quantification of Gzma^+^Ki-67^+^KLRG1^+^PD-1^−^ populations from (c). **e,** RV-Ctrl, RV-cMyc or RV-cMyc^T58A^ P14 cells were transferred individually into B-ALL-GP-bearing mice and survival was monitored. **f,** cMyc expression dynamics in human CD8^+^ T cells cultured in standard CAR-T cell expansion conditions. **g,** cMyc expression in human CD19-CAR and CD19-CAR-cMyc^T58A^. **h,** Expression of PD-1, CCR7, IL-2, IFNγ and TNF in CD19-CAR and CD19-CAR-cMyc^T58A^ T cells co-cultured with CD19^+^ Daudi cells. **i,** *In vitro* killing assay comparing human CD19-CAR T cells and CD19-CAR-cMyc^T58A^ T cells co-cultured with CD19^+^ Daudi cells. Graphs show mean ± S.E.M. from n = 4–6 mice (**c-e**) or pooled from >3 independent donors (**f-i**). *p < 0.05, **p < 0.005, ***p < 0.001, n.s. =non-significant, paired Student’s t-test or log-rank Mantel-Cox test.

Having established that cMyc GOF drives sustained metabolic fitness, stem-like properties, and resistance to terminal exhaustion in chronic viral infection, we next investigated whether these same programs could be engaged in cancer. Leveraging B-ALL-GP scGOF-seq data (Fig. 1i), we established that cMyc GOF increased P14 cell representation while concurrently enhancing stemness and reducing exhaustion signatures (Fig. 1m, o-p). Gene-set analysis further showed that cMyc GOF reshaped CD8^+^ T cell differentiation in B-ALL in a manner concordant with LCMV CL13, with enrichment of effector-associated programs and suppression of exhaustion and dysfunction signatures (Fig. 5a). Consistent with the chronic infection setting, cMyc GOF also induced coordinated transcriptional programs linked to metabolic fitness, proliferation, and anabolic activity (Fig. 5b).

To validate these effects at the cellular level, we performed three-way mixed transfers of control, cMyc GOF, and cMyc^T58A^ GOF P14 cells into B-ALL-GP bearing mice. As in chronic infection, both cMyc and cMyc^T58A^ GOF cells maintained elevated cMyc expression and reduced inhibitory receptor levels (Fig. 5c), consistent with scGOF-seq results (Fig. 1f-n). Both cMyc and cMyc^T58A^ GOF perturbations also increased Ki-67, GzmA, and KLRG1 expression, resulting in a ~150-fold expansion of a GzmA^+^Ki-67^+^KLRG1^+^PD-1^−^ effector-like population that was unique to Myc GOF and largely absent among control cells (Fig. 5d). Thus, cMyc GOF induced a conserved effector-featured, proliferative phenotype across chronic infection and leukemia.

We next asked whether these differentiation changes translated into therapeutic benefit. Adoptive transfer of cMyc GOF P14 cells significantly prolonged survival of B-ALL-GP bearing mice compared with controls (~50% survival), whereas the stabilized cMyc^T58A^ isoform further improved survival to ~80% (Fig. 5e). These data indicate that cMyc-driven programs identified during chronic infection can be repurposed to enhance T cell efficacy in leukemia.

**Extended Data Fig. 9 ∣:**
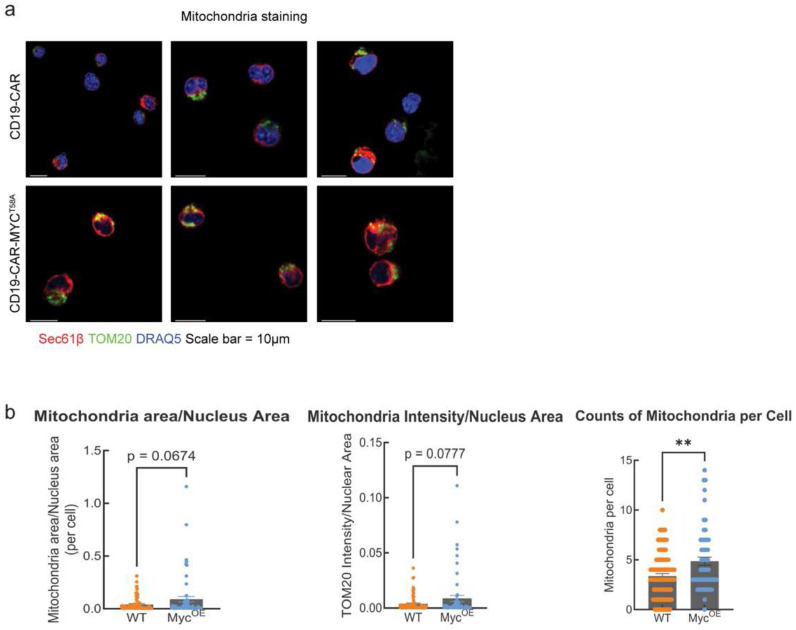
cMyc GOF increases mitochondrial mass in human CAR-T cells. **a,** Confocal microscopy of mitochondrial structure in human CD19-CAR-T cells or CD19-MYC^T58A^ CAR-T cells co-cultured in CD19+ Daudi cells. TOM20 is a mitochondria marker, SEC61β is an ER marker, and DRAQ5 is a nucleus marker. **b,** Quantification of mitochondrial counts and area from (a). Data are represented as mean ± SEM. Statistical analysis was performed using unpaired two-sided Student’s t test, **p < 0.01.

Given the magnitude of these effects, we examined whether cMyc GOF similarly reprograms human CAR-T cells. Under standard CAR-T cell expansion conditions with IL-2, IL-7, and IL-15^95^, activated human T cells showed transient cMyc induction between days 2 and 6, followed by downregulation to near-baseline levels (Fig. 5f), paralleling observations in mouse T cells (Fig. 2h; Extended Data Fig. 5d-f). We therefore introduced cMyc^T58A^ into an anti-CD19-CD28 CAR construct and co-cultured the resulting CAR-T cells with CD19^+^ leukemia cells (Fig. 5g). cMyc^T58A^-CAR-T cells showed increased IL-2, IFN-γ, and TNF-α production, reduced PD-1 expression, and increased mitochondrial mass (Fig. 5h; Extended Data Fig. 9a,b). They also exhibited significantly improved leukemia cell killing *in vitro* (Fig. 5i). Together, these findings suggest that sustained cMyc activity can enhance CD8^+^ T cell and CAR-T cell function in leukemia models and support the concept that temporally constrained GOF programs may be leveraged to improve cellular therapies.

## cMyc-induced stemness is insufficient for solid tumor control.

**Fig. 6 ∣:**
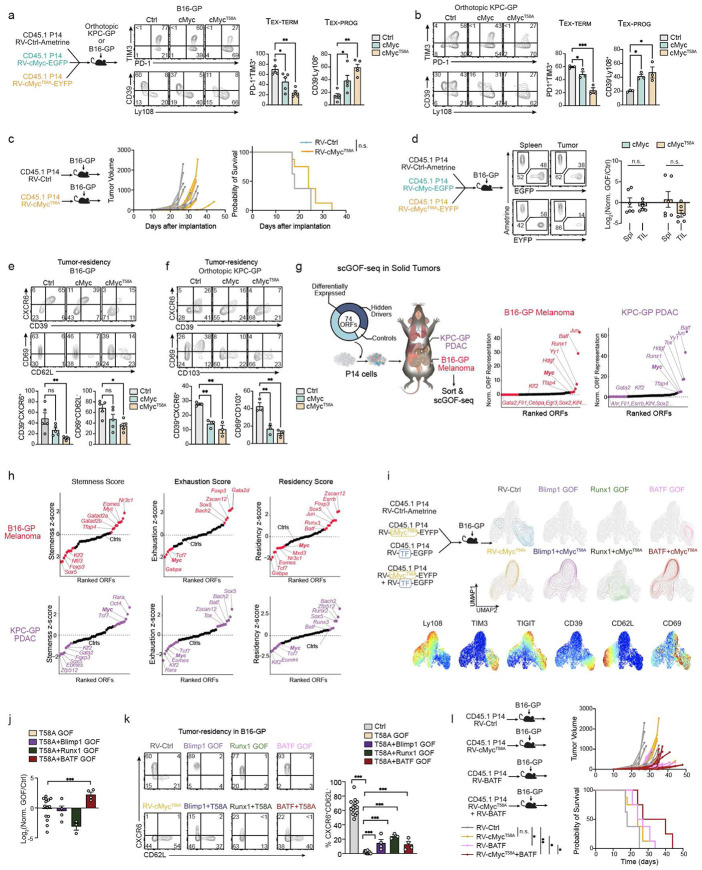
Rational GOF engineering enhances cell therapy efficacy in solid tumors. **a,b,** RV-Ctrl, RV-cMyc, or RV-cMyc^T58A^ P14 cells were mixed and transferred into mice bearing B16-GP (a) or orthotopic KPC-GP tumors (b). Tumors were harvested on days 8-12 days post-T cell transfer and cells were profiled by spectral flow cytometry. **c,** Tumor growth curves and survival of B16-GP–bearing mice receiving RV-Ctrl or RV-cMyc^T58A^ transduced P14 cells. **d,** Accumulation of RV-Ctrl, RV-cMyc, and RV-cMyc^T58A^ transduced P14 cells within B16-GP tumors from (a). **e,** Tumor-residency phenotypes of RV-Ctrl, RV-cMyc, and RV-cMyc^T58A^ P14 cells from B16-GP tumors (a). **f,** Tumor-residency phenotypes of RV-Ctrl, RV-cMyc, and RV- cMyc^T58A^ P14 cells in KPC-GP tumors from (b). **g,** scGOF-seq screening in B16-GP and orthotopic KPC-GP tumor models (left). Tumors were harvested 14 days after T cell transfer and normalized ORF representation was calculated for each model (right). **h,** Gene-module enrichment scores for individual ORFs from (g). **i,** Integrated analysis of combinatorial GOF perturbations assessed by spectral flow cytometry. Top, schematic of the *in vivo* combinatorial GOF strategy. CD45.1^+^ P14 cells transduced with RV-Ctrl, RV-cMyc^T58A^, single TF GOFs (Blimp1, RUNX1, or BATF), or dual GOFs combining each TF with cMyc^T58A^ were pooled and transferred into separate cohorts of B16-GP bearing mice. Cohorts included Ctrl + single cMyc^T58A^ GOF, Ctrl + single Blimp1 + dual GOF, Ctrl + single RUNX1 + dual GOF, and Ctrl + single BATF + dual GOF. Donor P14 cells recovered from all cohorts from day 10-11 post-transfer were analyzed via spectral flow. Bottom, UMAP projections of recovered donor P14 cells, colored by GOF identity. Feature plots show relative expression of differentiation and exhaustion markers (Ly108, TIM3, TIGIT, CD39, CD62L, CD69). **j,** Accumulation of normalized donor cells from (i). **k,** Phenotyping and quantification of tumor-resident populations by CXCR6^+^CD62L^−^ gating. **l,** B16-GP-bearing mice received 2x10^5^ P14 cells transduced with RV-Ctrl, RV-cMyc^T58A^, RV-BATF, or RV-cMyc^T58A^ + RV-BATF, and tumor growth was monitored longitudinally. Graphs show mean ± SEM of n=3-14 (a-l) from one representative experiment or pooled from two or more independent experiments. *p < 0.05, **p < 0.005, ***p < 0.001, n.s. =non-significant, paired Student’s t-test, unpaired Student’s t-test or log-rank Mantel-Cox test. For scORF-seq screens in (**g**), donor cells were pooled from n=8-10 mice.

Given that sustained cMyc expression enhanced ACT efficacy in B-ALL-GP and promoted CD8^+^ T cell stemness, we next investigated whether cMyc GOF could similarly improve adoptive cell therapy responses in solid tumors. We performed three-way mixed transfers of RV-Ctrl, RV-cMyc, and RV-cMyc^T58A^ P14 cells into tumor-bearing mice. We transferred donor cells into orthotopically implanted syngeneic pancreatic ductal adenocarcinoma tumor cells derived from C57BL/6 *Kras^G12D^Trp53^R172H^Pdx1-Cre* mice^96^ stably expressing the LCMV GP_33-41_ peptide (KPC-GP) or mice bearing B16-GP tumors (Fig. 6a-b). In both models, cMyc and cMyc^T58A^ GOF constrained terminal exhaustion and skewed differentiation towards Ly108^+^ TEX-PROG populations, with cMyc^T58A^ GOF yielding a ~2-4-fold increase relative to control. However, despite blunting of terminal exhaustion features and a reinforced stem-like phenotype, cMyc^T58A^ GOF cells failed to control tumor growth or improve survival in B16-GP (Fig. 6c). Thus, while cMyc GOF broadly enhances stemness under persistent antigen exposure, cMyc^T58A^ GOF was insufficient to confer therapeutic benefit in solid tumors.

**Extended Data Fig. 10 ∣:**
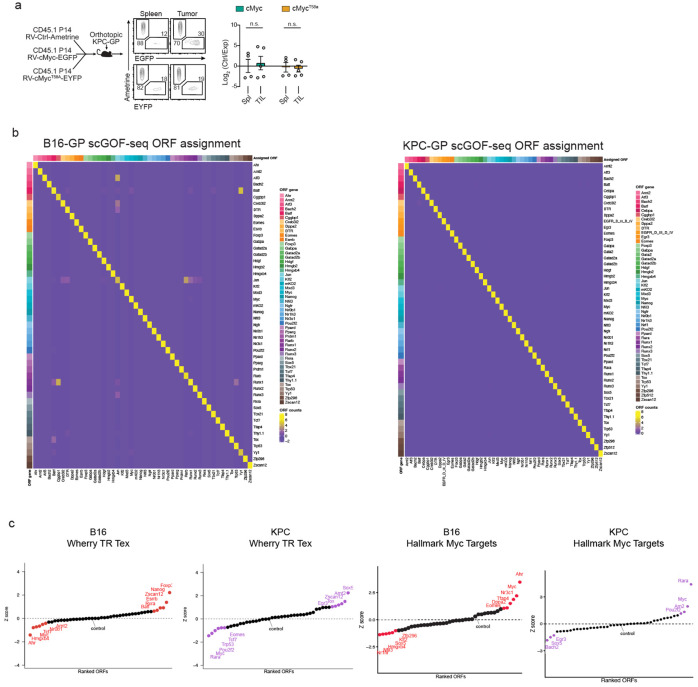
scGOF-seq in solid tumors. **a,** RV-Ctrl, RV-cMyc, or RV-cMyc^T58A^ P14 cells were mixed and transferred into mice bearing orthotopic KPC-GP tumors (from Fig. 6b). **b**, ORF assignment matrix for solid tumor experiments. Ranked ORF enrichment for relevant gene signatures.

**Extended Data Fig. 11 ∣:**
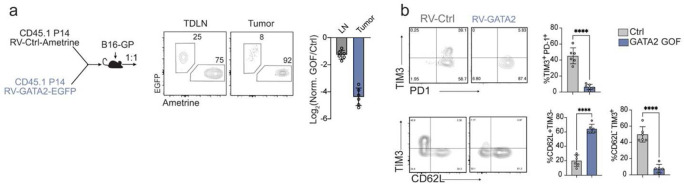
GATA2 GOF restrains TIL exhaustion and accumulation in a solid tumor model. **a,** Mixed transfer of RV-Ctrl and RV-GATA2 P14 cells into mice bearing B16-GP tumors. Relative accumulation in tumor-draining lymph nodes and tumors was evaluated day 12 post T cell transfer by spectral flow cytometry. **b,** Flow cytometric analysis and quantification of PD-1, TIM3, CD62L, CD69, and Ly108 expression in GATA2 GOF cells. Graphs show mean ± SEM of n=6 mice pooled from two independent experiments. ****p<0.0005, paired Student’s t-test.

Notably, cMyc GOF depleted tissue-residency gene expression programs (Fig. 3g) and failed to augment intestinal TRM state formation (Fig. 4c). Because tissue-residency programs are associated with enhanced CD8^+^ T cell adaption and retention in solid tumor environments^50,97^, we hypothesized that cMyc GOF failure in solid tumors may be due to impaired accumulation within the tumor microenvironment. Indeed, neither cMyc nor cMyc^T58A^ increased accumulation of donor T cells in B16-GP or KPC-GP models (Fig. 6d; Extended Data Fig. 10a). Consistent with this finding, cMyc GOF reduced expression of prototypical residency markers (CD69, CD103, CXCR6) while increasing lymphoid-homing CD62L expression within tumors (Fig. 6e-f). Multiomic analysis further suggested cMyc GOF limits activity of TFs supporting tissue-residency, including HIC1^98^, Blimp1^99,100^, and BATF ^50^ (Fig. 4k). We therefore leveraged scGOF-seq to overcome the limited efficacy of cMyc GOF in solid tumors to identify TFs that could complement molecular programs and phenotypes driven by cMyc GOF in solid tumors (Fig. 6g; Extended Data Fig. 10c). scGOF-seq screening of 74 TFs in B16-GP and KPC-GP tumors expectedly identified BATF and JUN as dominant drivers of P14 accumulation, whereas KLF2 reduced accumulation, consistent with prior work (Fig. 6g)^14,28,50,101^. scGOF-seq also captured expected roles for TCF1, RUNX3, and EOMES in regulating stemness and residency^9,50,102,103^, while uncovering unexplored regulators such as RUNX1 in enhancing TIL accumulation (Fig. 6g). Informed by scGOF-seq, we also found that hidden driver GATA2 blunts accumulation and features of exhaustion in tumor-specific T cells (Fig. 6g; validated in Extended Data Fig. 11a-b). Across both solid tumor models, scGOF-seq also demonstrated that cMyc GOF consistently promoted stemness while suppressing residency and accumulation (Fig. 6h), mirroring phenotypes observed in Fig. 6a-f.

## Combinatorial engineering integrates stemness and residency modules to drive superior efficacy in solid tumors.

We next tested whether rational combinations of GOF perturbations could be leveraged to enhance cell therapy responses in solid tumors. Based on scGOF-seq results, we paired cMyc^T58A^ with BATF and RUNX1 (TFs enhancing TIL accumulation from Fig. 6g) or Blimp1 (cMyc GOF antagonism identified from Fig. 4k-n) (Fig. 6i). While all combinations partially restored residency-associated features, only BATF GOF increased intratumoral accumulation of cMyc^T58A^ GOF cells (Fig. 6j) and promoted a CXCR6^+^ TRM-like state ^50,104^ (Fig. 6k). Functional testing revealed that BATF GOF improved tumor control^14,50,101^, and combining cMyc^T58A^+ BATF GOF improved tumor regression and survival benefit compared with RV-cMyc or RV-BATF cells (Fig. 6l). Collectively, these findings demonstrate that combinatorial GOF engineering, informed by scGOF-seq and exemplified by cMyc^T58A^ + BATF GOF, may be useful in supporting superior therapeutic outcomes in the context of cell therapies.

## Discussion

While LOF studies have defined essential regulators of T cell differentiation^9,10,13,14,105^, GOF perturbation provides a complementary strategy for uncovering regulatory activities that are constrained, silenced, or misplaced under physiological conditions^20-23^. Stable overexpression can extend TF activity beyond its endogenous temporal window, alter dosage or stoichiometry, and bypass feedback mechanisms that normally shape differentiation^106^. Here, we performed antigen-specific scGOF-seq across five immunocompetent disease models, demonstrating that enforced TF activity can modulate CD8^+^ T cell differentiation through multiple modes, including reinforcement or amplification of canonical programs, engagement of lineage-silent activities, and redirection of exhaustion-associated trajectories. Although ORF-based scGOF-seq is modestly less scalable than pooled CRISPR-based Perturb-seq approaches, it offers several advantages for this setting: it enables direct expression of full-length TFs independent of endogenous chromatin accessibility, avoids the large CRISPRa scaffolds that can be immunogenic in syngeneic *in vivo* models, and eliminates the need for multiple guide vectors per gene. Thus, scGOF-seq provides a complementary framework for mapping constrained regulatory potential that may not be apparent from endogenous expression patterns or LOF genetics alone.

A key insight from scGOF-seq was that lineage-silenced developmental TFs can exert measurable and context-dependent activities in mature CD8^+^ T cells when ectopically expressed. Factors such as NANOG, SOX2, OCT4, and GATA2 are not typically engaged during physiological T cell differentiation, yet their enforced expression reshaped exhaustion and stemness programs *in vivo*. These findings suggest that mature T cells retain access to regulatory responses not captured by endogenous expression or LOF studies. They also show that developmental identity alone does not predict functional output, as closely related pluripotency-associated TFs produced divergent effects on T cell accumulation, stemness, and exhaustion.

cMyc GOF exemplifies how enforced TF activity can reveal functions constrained by endogenous expression dynamics. cMyc is a canonical regulator of T cell activation, metabolism, and growth, and prior studies have linked exogenous cMyc^Hi^ states to effector commitment and limited self-renewal^33,35,107^. Consistent with these studies, endogenous cMyc^Hi^ CD8^+^ T cells in our experiments were associated with more terminally differentiated phenotypes. However, sustained cMyc expression outside its native temporal window produced a distinct outcome: stem-like differentiation coupled to effector features, metabolic fitness, reduced terminal exhaustion, and enhanced antigen-dependent expansion. Thus, the effects of cMyc GOF likely reflect not simply what cMyc does in physiologic T cell differentiation, but what sustained cMyc activity can reveal when its expression is uncoupled from normal temporal control.

cMyc-induced a unique effector-like state (Tex-myc^Unique^) that was distinct from conventional Tex-int, Tex-klr and Tex-eff populations, and exhibited enhanced proliferative, translational, and metabolic activity. This distinction suggests that sustained cMyc GOF does not merely expand an existing effector-like exhausted subset but uncouples effector-associated features from exhaustion-linked metabolic impairment. In contrast to increased translation in exhausted TILs driven by GADD34-mediated p-eIF2α attenuation or ATF4 upregulation, which has been associated with dysfunctional states^108,109^, the anabolic programs induced by cMyc GOF were accompanied by proliferative fitness and cytokine production. Functionally, restoring Blimp1 activity opposed major components of the cMyc GOF phenotype, consistent with roles of Blimp1 in repressing mitochondrial fitness, IL-2 production, and proliferation^90,92,110^. Together, these findings support a model in which cMyc GOF reconfigures multiple coupled dimensions of exhaustion, including metabolism, proliferation, and cytokine production, rather than simply reversing exhaustion through a single linear pathway.

cMyc GOF also operated in a strongly context-dependent manner. Despite the proto-oncogenic potential of cMyc^111^, enforced cMyc or cMyc^T58A^ expression did not produce ubiquitous or antigen-independent expansion in the settings tested. Accumulation was modest during acute infection, absent in some non-lymphoid compartments, and dependent on persistent antigen. Long-term follow-up and transfer into irradiated hosts lacking cognate antigen did not reveal autonomous outgrowth, and cMyc GOF cells retained cell-cycle checkpoint signatures without inducing selected cMyc-associated malignant programs. These observations do not eliminate the need for long-term safety evaluation, particularly in therapeutic settings, but they indicate that cMyc-driven expansion of mature CD8^+^ T cells remains constrained by antigen availability in the models examined. Future therapeutic implementation of potent GOF programs will likely require conditional control strategies, including tunable expression systems, suicide switches, or other approaches for regulating transferred cells^112-116^.

The context dependence of cMyc GOF was most evident in solid tumors. Although cMyc enhanced stemness and metabolic fitness, these features were insufficient for robust solid-tumor control, likely because effective tumor immunity requires coordination of additional programs governing tumor-residency, survival, and effector differentiation. scGOF-seq identified BATF as a cooperating factor that complemented cMyc-driven programs and improved solid-tumor responses. This interaction illustrates how single-factor GOF perturbations can reveal useful but incomplete regulatory modules, whereas combinatorial perturbations can assemble more effective T cell states by integrating complementary programs. These findings are consistent with recent work identifying the cMyc effector AP-4 as a regulator of T cell fitness that can pair with BATF GOF^24^, and support broader efforts to rationally combine GOF and LOF perturbations to reprogram T cell fate^10,25,105^.

Finally, many current immunotherapy strategies focus on releasing inhibitory brakes, such as blocking PD-1 signaling or suppressing exhaustion-associated TFs. Our work supports a complementary strategy: identifying and engaging constrained regulatory activities that enhance T cell persistence, fitness, and function. Integrating scGOF-seq with multi-omics-guided TF discovery, network inference, and predictive modelling could enable the design of minimal TF combinations tailored to specific disease contexts. Together, these findings position GOF perturbation as a framework for mapping constrained regulatory potential in immune cells and guiding rational engineering of therapeutic T cell states.

## Methods

### Mice

All mice were bred and housed in specific pathogen-free conditions on a 12-hr light-dark cycle at ambient temperature in accordance with Institutional Animal Care and Use Guidelines of the University of North Carolina, Chapel Hill. CD45.1 or CD45.2 C57BL/6 mice aged between 6 and 18 weeks old were used as recipients for cell transfer experiments. Transgenic TCR (P14) mice that recognize the LCMV peptide GP_33-41_ were gifted by Dr. Ananda Goldrath (UCSD). P14 cMyc^GOF-Tg^ mice were generated by crossing dLck-Cre^117^ P14 mice to Gt(ROSA)26 Sor^tm13(CAG-MYC,-CD2*)Rsky^/J mice^118^ obtained from the Jackson Laboratory (JAX strain 033805). P14 ERT2-Cre h*MYC*-h*CD2*^LSL^ (iCre cMyc^GOF-Tg^) mice were generated by crossing ERT2-Cre P14 mice with Gt(ROSA)26 Sor^tm13(CAG-MYC,-CD2*)Rsky^/J mice. Mice with EGFP fused to the 3’ end of cMyc exon 3 (Myc^tm1.1Dlev^/J, strain 019075)^65^ were obtained from JAX, then crossed to P14 transgenic mice. Mice were bred in-house and fed standard Purina chow. Donor and recipient mice were either sex-matched or female cells were transferred into male mice. Both male and female mice were used.

### T cell transfers, LCMV infection, and treatments

For adoptive transfer of T cells, 2.5-5.0 x 10^3^ naïve P14 cells were transferred into congenially distinct recipient mice one day prior to intravenous infection with 2-4x10^6^ PFU LCMV CL13 or 2-3x10^4^ naive P14 cells were transferred one day prior to intraperitoneal infection with 2x10^5^ PFU LCMV Arm.

For adoptive transfer of transduced T cells, negatively enriched CD8^+^ T cells were first isolated using the EasySep Mouse CD8^+^ T cell Isolation Kit (StemCell, cat# 19853A) following manufacturer’s instructions, then activated in wells coated with 100 μg/mL goat anti-hamster IgG (H+L; Invitrogen), 1 μg/mL plate-bound anti-CD3 (145-2C11; eBioscience), and 1 μg/mL anti-CD28 (37.51; eBioscience). Approximately 18-24h after activation, CD8 T cells were transduced with retroviral vectors as described previously^50^. One day after transduction, transduced T cells were mixed, input ratio was measured, and 1-5 x 10^5^ cells were transferred into congenic mice infected with LCMV. Retroviral vectors used for these studies are outlined in Table S1.

For sort transfer experiments, PD-1^+^CX3CR1^+^Ly108^−^KLRG1^−^ CD8^+^ T cells were sorted from LCMV CL13 infected iCre cMyc^GOF-Tg^ mice 20 days post-infection, and 5 x10^4^ donor cells were transferred to infection matched recipient mice. Recipient mice were treated with 200 μg of anti-CD4 (GK1.5; BioXcell) the day before and day following LCMV CL13 infection to deplete CD4 T cells for deeper T cell exhaustion. Tamoxifen-induced activation of cMyc GOF was achieved by intraperitoneal injection of 16.7 μg/mL tamoxifen diluted in sunflower oil 24 and 48 hours post transfer of sorted cells. Transferred CD45.2 iCre cMyc^GOF-Tg^ cells were profiled 12 days post transfer via spectral flow cytometry.

### Tumor models

All cell lines were maintained in sterile culture conditions at 37°C and 5% CO_2_, at or below 80% confluency, and were routinely tested for Mycoplasma. All tumor cell lines (B16-F10 melanoma (obtained from Dr. Ananda Goldrath), KPC-4662 pancreatic ductal adenocarcinoma (obtained from Dr. Robert Vonderheide), and B-ALL leukemia (obtained from Dr. Martine Roussel) used in this paper express the LCMV GP_33-41_ peptide and were previously generated and validated^50^. B16-GP and KPC-GP cell lines were grown in complete DMEM with 10% FBS. The B16-GP growth media was supplemented with 300 μg/mL of geneticin. B-ALL-GP cells and Daudi cells (obtained from Dr. Gianpietro Dotti (UNC-CH) were grown in complete RPMI with 10% FBS.

For B16-GP experiments, 0.5-1 x 10^6^ B16-GP cells were implanted subcutaneously into the flank 7-10 days before adoptive transfer of 1-2 x 10^6^ activated and expanded P14 T cells. P14 T cells were expanded with 20 U/mL rIL-2 (Peprotech), 2.5 ng/mL rIL-7 (Peprotech), and 2.5 ng/mL rIL-15 (Peprotech) for 2-4 days prior to transfer to congenic, tumor-bearing mice. For P14 cell efficacy experiments, 7.5 x 10^5^ B16-GP cells were implanted and 2.5 x 10^5^ transduced P14 T cells were transferred on the same day of transduction to maximize engraftment. Mice were euthanized if tumors ulcerated or reached 20mm in any direction. For B-ALL-GP phenotyping experiments, mice were inoculated intraperitoneally with 1.5 x 10^4^ B-ALL-GP cells one day before intravenous transfer of 2 x 10^3^ activated and transduced P14 T cells. For B-ALL-GP efficacy experiments, 5 x 10^3^ B-ALL-GP cells were implanted and 2 x 10^4^ transduced P14 T cells were transferred the following day. For experiments with KPC-GP tumor-bearing mice, 1.5-2.5 x 10^5^ KPC-GP cells were surgically implanted orthotopically into the tail of the pancreas as described previously^50^. 7-10 days after KPC-GP implantation, 1-2 x 10^6^ transduced P14 T cells were adoptively transferred.

### Tissue processing, spectral flow cytometry, and cell sorting

Single-cell suspensions from spleens were obtained through mechanical disruption and RBCs were lysed with ACK buffer (150 mM NH4Cl, 1mM KHCO_3_, and 0.1 mM EDTA, pH 7.4). Intraepithelial lymphocytes (IELs) were obtained from small intestines through chemical digestion in a buffer containing 154 μg/mL DTE for 30 min at 37°C following removal of Peyer’s patches. Tumor infiltrating lymphocytes were obtained from tumors through mechanical disruption and chemical digestion in RPMI with either 100 IU/mL Collagenase I (Worthington), 1mM MgCl2, 1mM CaCl2, 1% HEPES, 1 mM L-glutamine, and 5% bovine growth serum for B16 tumors or 1.25 mg/mL Collagenase IV (Worthington), 1 mg/mL DNAse I (Worthington), 0.1% Trypsin Inhibitor from Soybean (Worthington), and 5% bovine growth serum for KPC tumors for 30 min at 37°C. Following digestion, single-cell suspensions of lymphocytes were obtained from tumor and small intestines by filtering cells through a 70-μm nylon filter and then density gradient spin isolation was performed using 44/67% Cytiva Percoll Centrifugation Media (Fisher Scientific, cat# 45-001-747) for 20 min at 568g.

For flow cytometry analysis, all samples were stained with fixable live/dead dye (Invitrogen) and FcR block (S17011E; Biolegend) prior to further staining. The H-2D^b^-GP_33-41_ and H-2D^b^-GP_276-286_ tetramers were obtained from the National Institutes of Health Tetramer Core. The following antibodies were used in flow staining and purchased from Biolegend, unless otherwise noted: CD8α (53-6.7), CD8β (YTS156.7.7), CD45.1 (A20), CD45.2 (104), Tim3 (RMT3-23), Ly108 (330-AJ and 133G3; BD Biosciences), PD-1 (29F.1A12), TIGIT (1G9), CX3CR1 (SA011F11), CD62L (MEL-14), CD103 (2E7), CD69 (H1.2F3), CD127 (A7R34), CD44 (IM7), anti-human CD2 (TS1/8), Lag3 (C9B7W), CXCR6 (SA051D1), CD39 (Duha59), KLRG1 (2F1; Invitrogen), CD101 (Moushi101; Invitrogen), puromycin (2A4), IL-2 (JES6-5H4), IFN-γ (XMG1.2), TNF (MP6-XT22), GzmB (QA16A02), GzmA (3G8.5), BATF (S39-1060; BD Biosciences), TOX (REA473; Miltenyi Biotec), Ki67 (SoLA15; Invitrogen), TCF1 (C63D9; Cell Signaling), cMyc (E5Q6W; Cell Signaling), and anti-rabbit IgG (polyclonal; Cell Signaling). Intracellular staining was performed with the FoxP3 Transcription Factor Staining Kit (Invitrogen) according to manufacturer’s instructions following 5-8min fixation with 2% PFA at room temperature.

For metabolic assessment of CD8^+^ T cells by flow, cells were treated with uptake and mitochondrial dyes as described previously^119^. In brief, cells were incubated with 15 nM Mitotracker Deep Red (ThermoFisher) and 100 nM Mitotracker Red CMXRos (ThermoFisher) for 10 min at 37°C. Cells were then washed in cold PBS prior to extracellular staining. Alternatively, cells were incubated with 0.4 μM glucoseCy5 (Sigma Aldritch) for 30 min at 37°C. Cells were then washed in cold PBS before extracellular staining. Protein translation was assessed as similarly described in the SCENITH assay^75^, where Puromycin was introduced into media of ex vivo cultured CD8^+^ T cells for 30 min prior to staining of cells and intracellular staining of puromycin followed by spectral flow cytometry analysis.

All flow cytometry analysis was performed on a 5L Cytek Aurora spectral flow cytometer. All cell sorting was performed on a BD Aria II or Sony SH800 sorter. For LCMV sorts, CD4 T cells, RBCs, B cells were depleted from cell preparations on Miltenyi Biotec magnetic columns using biotin conjugated antibodies, CD4 (GK1.5), CD45R/B220 (RA3-6B2), CD19 (8D5), and TER-119 (TER-119), and streptavidin conjugated magnetic beads prior to sorting.

### Human CAR-T cell studies

#### Human CAR-T cell preparation.

Human CAR-T cells were generated from peripheral blood mononuclear cells (PBMCs) isolated from fresh human blood (Gulf Coast Regional Blood Center). PBMCs were resuspended in complete CAR-T medium (1:1 RPMI 1640: Click’s medium supplemented with 10% FBS, GlutaMAX, penicillin/streptomycin, and 0.05 mM 2-mercaptoethanol) and activated on non–tissue culture–treated 24-well plates pre-coated with anti-human CD3 (Cytek) and anti-human CD28 (BD Pharmingen) monoclonal antibodies (1 μg/mL each). One day after activation, IL-7 (10 ng/mL) and IL-15 (5 ng/mL) were added. On day 2 post-activation, T cells were transduced with either control CD19.CAR or cMycT58A.CD19 CAR retroviral supernatant on retronectin-coated 24-well plates: wells were coated overnight with retronectin (7 μg/mL), loaded with viral supernatant, and centrifuged at 2,000×g for 90 min at 32°C before adding 5e5 activated T cells per well in cytokine-containing CAR-T medium, followed by centrifugation at 1,000×g for 10 min at 32°C. Cells were then cultured at 0.5-1 x 10^6^ cells/mL in IL-7/IL-15 supplemented CAR-T cell media, with media and cytokines refreshed every 2–3 days. CAR-T cells were used for experiments between days 8 and 12 post-activation.

#### In vitro cancer killing assay.

Co-culture of CAR T cells and Daudi cancer cells was performed as previously described^24,120^. Daudi cells were seeded in 96-well plates at 1e5 cells/well and co-cultured at a 1:1 ratio with either control CD19.CAR T cells or cMycT58A.CD19 CAR T cells. Cells were cultured for 6 days in the presence of 100 IU/mL rhIL-2 before assessment of T cell exhaustion and cytokine production by flow cytometry.

#### Mitochondria and ER confocal microscopy of human CAR-T cells.

CD8+ CAR-T cells were sorted on viable CD8+/GS4 (CAR marker)^+^ gates. Cells were blocked with 2% BSA in PBS for 1 hour at room temperature before antibody staining with rabbit α-Sec61b (Cell Signaling) and mouse α-Tom20 (Santa Cruz) antibodies in 1% BSA in PBS for 1 hour at room temperature. Cells were washed with 1x PBS and incubated with nuclear stain DRAQ5 (Abcam), and secondary antibodies goat anti-rabbit IgG (H+L) AlexaFluor 594 and goat anti-mouse IgG (H+L) AlexaFluor 488 (ThermoFisher) in 1% BSA in PBS for 1 hour at room temperature. A 96-well glass bottom plate (Cellvis) was coated with CellTak (Corning), washed with DI water, air dried, and labeled cells were centrifuged for adhesion. Cells were imaged using the Zeiss LSM-880 confocal microscope with ZEN acquisition software (Zeiss) or AiryScan detector and 3-D reconstruction was generated from confocal z-stacks using Imaris software. All quantitation indicates a per-cell measurement (i.e., a single point in the Mitochondria area/Nucleus area plot refers to the area of positive TOM20 signals above a manually-defined threshold, divided within a single cell, divided by the area of the nucleus (DRAQ5 positive signal from a manually-defined threshold) for that same cell. The threshold for both TOM20 and DRAQ5 signals was identical for segmentation for both samples. Manually-identified cells with irregular morphology or appearance representative of an apoptotic cell were excluded from calculations.

### Retroviral Transductions

All mouse CD8^+^ T cell transductions were performed as described previously^53^ using packaged MSCV retrovirus, supplemented with 8 μg/mL polybrene and 50 μM BME, via spin-fection for 1 hr at 37°C and 568g. In addition to vectors included in the ORF screen (Table S1), the following vectors were obtained from Vector Builder, Addgene, or subcloned with the In-Fusion cloning approach: cMyc-IRES-EYFP (Vector Builder), cMyc^T58A^-IRES-EGFP (Addgene; cat#177648), cMyc^T58A^-IRES-EYFP (Vector Builder), and IRES-Ametrine (Vector Builder). Retrovirus was generated as previously described^53^. In brief, PLAT-E retrovirus packaging cells were plated with 5e6 cells per well of 6-well plate. 24-hrs later, or once cells reached 70% confluency, Plat-E were treated with TransIT-LT1 transfection reagent mixed with 1.5 μg of vector plasmid and 1 μg pcl-eco helper plasmid (1.5:1 ratio) at a 3:1 ratio of TransIT-LT1 to DNA. Retrovirus was harvested 48 and 72h following transfection.

### scGOF-seq Library Preparation and Sequencing

#### scGOF-seq screening library and sample preparation.

All plasmids for the ORF screen were obtained from VectorBuilder on an MSCV backbone with an EGFP reporter (Table S1). Enriched and activated P14 CD8^+^ T cells were transduced with retrovirus in an arrayed format. For LCMV screens, 3e5 pooled cells were transferred into recipient mice one day after infection with LCMV Arm or LCMV CL13. On day 14 of infection EGFP^+^ donor cells were sort purified. For B-ALL-GP screening, 2.5e5 pooled P14 T cells were transferred to recipient mice inoculated with 1.5e4 B-ALL-GP cells one day prior. For B16-GP screening, 1e6 pooled P14 T cells were transferred into mice bearing B16-GP cells implanted 10 days prior to T cell transfer. For KPC-GP samples, 2e6 pooled cells were transferred into mice bearing orthotopically implanted KPC-GP cells 10 days prior to T cell transfer. For all screens, EGFP^+^ donor CD8^+^ T cells were sorted from either splenocytes (LCMV Arm, LCMV CL13, B-ALL) or tumor (B16, KPC) on a Sony SH800 sorter. B-ALL-GP samples were sorted 10 days after T cell transfer, B16-GP samples and KPC-GP samples were sorted 14 days after T cell transfer.

#### LCMV Arm and CL13 scGOF-seq library preparation.

Gene expression libraries were prepared using the Chromium Next GEM Single Cell 5’ Reagent Kit v2 (Dual Index) following manufacturer instructions. Custom primer sequences and concentrations used throughout the process are listed in Table S2. For ORF library preparation, reverse transcription reactions were spiked with a custom reverse primer “EGFP Rev 1” in addition to the standard poly-dT RT Primer to ensure efficient capture of EGFP transcripts (at a 1:6.3 ratio). For cDNA library amplification of LCMV Arm and CL13 libraries, and the entire volume of reverse transcribed cDNA was amplified 12 cycles using 12.9uL of the standard 10x Genomics cDNA Primer Mix as well as 2.1uL of an EGFP-specific “EGFP Rev for cDNA” spike-in reverse primer. Libraries were prepared from amplified cDNA products as described in the 10x Genomics user guide. In addition, ORF transcript enrichment was performed following a slightly modified version of the 10x V(D)J enrichment procedure. The standard 10x forward primer (“CommonFwd”) and custom outer and inner reverse primers (“EGFP Rev2” and “EGFP Rev3”, respectively) were used in place of the V(D)J primer mixes. Arm and CL13 gene expression libraries were sequenced at the UNC High Throughput Sequencing Facility using one lane of a NovaSeq 6000 S4 flow cell (R1: 26. i1: 10, i2: 10, R2: 90). ORF libraries were sequenced at Admera Health using one lane of a NovaSeq X Plus 10B flow cell without standard 10x read trimming (R1: 150, i1: 10, i2: 10, R2: 150). FASTQ filename prefixes indicate the library type (descriptions available in Table S3).

#### Cancer scGOF-seq library preparation.

Gene expression libraries were prepared using the Chromium Next GEM Single Cell 5’ Reagent Kit v2 (Dual Index) following manufacturer instructions. Custom primer sequences and concentrations used throughout the process are listed in Table S2. B-ALL, B16, and KPC samples were processed as described for LCMV samples above with a few alternations to the protocol. The reverse transcribed cDNA was split into two separate cDNA amplification reactions: 60% was amplified as described above, and 35% was amplified using only EGFP specific primers (“FWD for cDNA” and “EGFP Rev PCR”). Next, we followed a standard ORF libarary preparation with a nested amplification of EGFP-containing cDNA molecules. Reaction conditions and post-cycling cleanup steps followed the standard V(D)J protocol. All amplifications were 12 cycles. Gene expression libraries and ORF libraries were sequenced at Admera Health using one lane of a NovaSeq X Plus 10B flow cell without standard 10x read trimming (R1: 150, i1: 10, i2: 10, R2: 150). FASTQ filename prefixes indicate the library type (descriptions available in Table S3).

#### Sequencing data processing and ORF assignment.

Barcodes and UMIs from single-cell ORF FASTQs were extracted and appended to the read name via fastp (v0.23.4). Forward and reverse reads were independently aligned to a custom reference genome using bwa-mem2 (v2.2.1) invoking the option to output all reads (-a) followed by samtools (v1.20). The reference genome contained relevant ORF sequences for each of the experiments (Cancer – B-ALL/B16/KPC or LCMV), as well as upstream and downstream vector sequences (LTR 5′ region, MSCV Kozak region, T2A, and EGFP, as well as Malat1, Supplementary data file 1). Vector-derived ORF sequences in the reference genome are exclusively exonic in nature. All single-cell FASTQs (ORF and GEX) were processed via 10x Genomics Cell Ranger count v 7.1.0. Reads were aligned to a custom reference genome consisting of 2024-A (Mouse GRCm39 – GENCODE vM33) plus additional vector sequences and exogenous sequences (DTR, EGFRd/ EGFR_D_III_D_IV, mKO2, Ngfr (cancer data only) and Thy1.1 (cancer data only). To account for the fact that endogenous RNA sequence was present at high levels in the ORF libraries as measured by reads aligning to Malat1, custom python scripts were utilized to process bam files from bwa-mem2 and derive read counts for each ORF and cell. After filtering for MAPQ >=20, reads were counted as ORF-derived if they met one of two conditions: 1) R1 aligned to vector sequence and R2 aligned to ORF sequence (or vice versa) or 2) they had a primary and supplementary alignment combination that transversed the ORF and upstream or downstream vector sequence. In the case of the latter condition, only reads with congruent information from R1 and R2 were used. Corrected cell barcodes from the Cell Ranger bam files were used to correct any barcodes from the bwa alignments. Finally, cells were assigned to an ORF if they met one of two conditions: 1) had at least 6 reads from a single ORF, or 2) had more than 50 reads from a single ORF.

#### Processed scGOF-seq data analysis.

Cells from each sample were processed independently via the Seurat package (v5.1.0) in R (v4.3.3). Data were filtered to exclude any cells with more than 12% of reads derived from mitochondrial sequences, fewer than 3000 UMI counts, and fewer than 300 genes. Data were normalized via standard LogNormalization, and 3000 variable features were used for PCA dimension reduction. The first 30 principal components were used as input for neighbor graph construction and UMAP dimension reduction. Clustering was performed at multiple resolutions via the Louvain algorithm with multi-level refinement.

Input cell proportions were used to calculate ORF enrichment by normalizing ORF frequencies in experimental samples to their corresponding ORF and control proportions in the input donor cell population. ORF outputs normalized by input are available in **Data S1**. Enrichment scores were ranked and visualized accordingly. To generate ORF density plots, we used Nebulosa^121^ to recover and smooth signals from sparse features in single-cell datasets. gRNA density plots were generated using scCustomize^122^, which applies kernel density estimation to visualize guide RNA distributions across cell states. Gene signature log2 foldchange comparison between Ctrl vs cMyc GOF in Fig. 4A was performed using the R package escape^123^ (2.6.1). Gene signature and metabolic pathway enrichment were quantified using the R package UCell (v2.2), which computes enrichment scores based on the Mann–Whitney U statistic^124^. UCell scores for each ORF were Z-score normalized and visualized as ranked enrichment profiles. Summary of scGOF-seq hits is available in **Data S2**. Gene sets used in this study is available in **Data S3**.

### cMyc^GOF-Tg^ *vs*. WT scRNA Sequencing and analysis

cMyc^GOF-Tg^ or littermate WT mice (*dLckCre*^+/−^) were infected with 4 x 10^6^ PFU LCMV CL13 intravenously. CD44^+^ CD8^+^ T cells were sorted from splenocytes on D7 and D15 post-infection on a Sony SH800 sorted. D15 samples were prepared following manufacturer instructions for the Chromium Next GEM Single Cell 3’ Reagent Kit v3.1 (Dual Index). D7 samples were labeled with 10x Genomics CellPlex cell multiplexing oligos, pooled, and prepared following manufacturer instructions for the Chromium Next GEM Single Cell 3’ Reagent Kit v3.1 (Dual Index) with Feature Barcode Technology for Cell Multiplexing. Libraries were sequenced at the UNC High Throughput Sequencing Facility using a NextSeq 2000 P3 flow cell (R1: 28, i1: 10, i2: 10, R2: 90). Gene signature enrichment comparison between Ctrl vs Tex-myc^UNIQUE^ or Ctrl vs cMyc GOF in Fig. 2N was performed using the R package escape^123^ (2.6.1). Gene sets used in this study is available in **Data S3**.

### Bulk ATAC-seq

cMyc^GOF-Tg^ or littermate WT mice (*dLckCre*^+/−^) were infected with 4x10^6^ PFU LCMV CL13 intravenously. CD44^+^ PD-1^+^ CD8^+^ T cells were sorted from mice 15 days post-infection on a BD FACS Aria II. Samples were prepared following manufacturer instructions using the Active Motif ATAC-Seq kit (Cat# 53150), with each sample receiving a unique combination of i7 and i5 barcodes. Samples were pooled for sequencing on a NovaSeq X Plus 10B flow cell at Admera Health. ATAC processing was done in command line using Bioconda packages: macs2 (v 2.2.9.1), fastqc (v 0.12.1), bwa (v 0.7.18), picard (v 3.1.1), samtools (v 1.19), bedtools (v 2.31.1), and trimmomatic (v 0.39). Adapter sequences were trimmed from reads, using trimmomatic, before mapping to the mm10 reference genome and generating bed files for peak calling, using picard, samtools, and bedtools.

### Taiji multiomics-based TF analysis

#### Identification of differentially active, less variable, and low-expression TFs.

To analyze TF activity and expression in fig. S1, we performed an integrative analysis of single-cell multiomics data^50^ using Taiji v2.0, as we recently described^83^. Counts per million (CPM) of pseudobulk raw gene expression counts were used to calculate the TF activity score (PageRank). Next, we compared the median PageRank across 16 cell states and calculated coefficients of variation (CV) to assess the specificity of each TF in each cell state.

#### cMyc regulatory networks in cMyc GOF vs. WT construction.

Differential TF activity in cMyc GOF vs. WT CD8+ T cells was analyzed with Taiji v2.0, integrating scRNAseq and bulk ATAC-seq data from cMycGOF-Tg vs. WT, as described previously^104^. TF activity and log2FC were calculated in each context. Subsequently, a cMyc-TF correlation matrix was generated by calculating Spearman’s correlation of edge weights for each TF pair across their common regulatees. From this matrix, we constructed a graphical model using the R package “huge”^125^, which employs the Graphical Lasso algorithm and a shrunken ECDF (empirical cumulative distribution function) estimator. An edge between two TFs was established if their correlation was deemed significant by the model, with a lasso penalty parameter (lambda) of 0.052.

### Signature enrichment analysis comparing cMyc-induced lymphoma and cMyc-transgenic lymphocytes

To compare the enrichment of gene signatures associated with cMyc-induced malignancy versus cMyc-transgenic non-malignant lymphocytes (Fig. 4j; Extended Data Fig. 8b), we analyzed two datasets: RPKM-normalized bulk RNA-seq data from Sabo et al^79,^ utilizing Eμ-*myc* transgenic mouse, containing the mouse cMyc oncogene linked to an immunoglobulin heavy-chain enhancer Eμ and the relative count-normalized B-ALL-GP33 scGOF-seq dataset. For the Sabo et al. dataset, we additionally performed within-gene normalization to better visualize differences among non-Eμ-*myc* transgenic control lymphocytes, Eμ-*myc* normal lymphocytes and Eμ-*myc* lymphoma samples. Using the differential expression results reported by Sabo et al. for Eμ-*myc* normal lymphocytes versus control and Eμ-*myc* lymphoma versus control comparisons, we defined two gene signatures. Eμ-*myc* normal UP and Eμ-*myc* Lymphoma UP **(Extended Data Table 3)**. Signature enrichment scores were calculated for both datasets using UCell in R. Scores were visualized by sample type for the bulk RNA-seq dataset and by cell state for the scGOF-seq dataset.

### Statistical analyses

For comparisons between two groups, two-tailed unpaired or paired t-tests were performed, with Welch’s correction as necessary for significantly different variances. When comparing multiple groups, one-way ANOVA with Tukey’s multiple comparison’s test was performed. P values < 0.05 were considered significant. Unless otherwise noted, error bars are standard error of mean and significance is noted as follows: *, p ≤ 0.05; **, p ≤ 0.01; ***, p ≤ 0.001.

#### Statistical analyses:

Statistical tests for flow cytometry data were performed using Graphpad Prism 10. For comparisons between two groups, two-tailed unpaired or paired t-tests were performed, with Welch’s correction as necessary for significantly different variances. When comparing multiple groups, one-way ANOVA with Tukey’s multiple comparison’s test was performed. P values < 0.05 were considered significant. Unless otherwise noted, error bars are standard error of mean and significance is noted as follows: *, p ≤ 0.05; **, p ≤ 0.01; ***, p ≤ 0.001.

## Extended Data

## Supplementary Material

Supplement 1 Supplementary Information is available for this paper. Correspondence and requests for materials should be addressed to H. Kay Chung (hkchung@unc.edu), J. Justin Milner (justin_milner@med.unc.edu).
This is a list of supplementary files associated with this preprint. Click to download.
EDtable3.Gensetsusedinthisstudy.xlsxEDTable2.SummaryofscGOFseqhits.xlsxEDTable1.InputrepresentationandnormalizedcellrecoveryfromscGOFseqscreens.xlsx

## Data Availability

All data and code are available upon request. All sequencing datasets generated for this study have been deposited at GEO: GSE302609, GSE302580, and GSE309695 (scGOF-seq) and will be made publicly available as of the date of publication. Information and requests for reagents, code, and data should be directed to the lead contacts, H. Kay Chung (hkchung@unc.edu) or Justin Milner (justin_milner@med.unc.edu). All materials will be made available upon request.

## Code availability

Custom scripts used for ORF assignment and downstream analyses will be made available through GitHub upon publication.
