# Supplementary material for: Single-cell gain-of-function mapping reveals latent regulatory programs governing CD8+ T cell fate
Source: Res Sq. 2026 Jun 11:rs.3.rs-9929244. Preprint. [Version 1] doi: 10.21203/rs.3.rs-9929244/v1 (PMC13278306; doi:10.21203/rs.3.rs-9929244/v1)
Supplement: Supplement 1 [file NIHPPrs9929244v1-supplement-1.pdf]

Table S1

Gene	scGOF-screen			
Ahr		B-ALL-GP	B16-GP	KPC-GP
Arnt2		B-ALL-GP	B16-GP	KPC-GP
Atf3	LCMV	B-ALL-GP	B16-GP	KPC-GP
Bach2	LCMV	B-ALL-GP	B16-GP	KPC-GP
Batf	LCMV	B-ALL-GP	B16-GP	KPC-GP
Bcl6	LCMV	B-ALL-GP	B16-GP	KPC-GP
Cebpa	LCMV	B-ALL-GP	B16-GP	KPC-GP
Cggbp1		B-ALL-GP	B16-GP	KPC-GP
Creb3l2		B-ALL-GP	B16-GP	KPC-GP
Crem	LCMV	B-ALL-GP	B16-GP	KPC-GP
Ctcf		B-ALL-GP	B16-GP	KPC-GP
Dppa2		B-ALL-GP	B16-GP	KPC-GP
DTR	LCMV	B-ALL-GP	B16-GP	KPC-GP
EGFR D III – D IV	LCMV	B-ALL-GP	B16-GP	KPC-GP
Egr3	LCMV	B-ALL-GP	B16-GP	KPC-GP
Eomes	LCMV	B-ALL-GP	B16-GP	KPC-GP
Esrrb	LCMV	B-ALL-GP	B16-GP	KPC-GP
Fli1		B-ALL-GP	B16-GP	KPC-GP
Foxp3	LCMV	B-ALL-GP	B16-GP	KPC-GP
Gabpa		B-ALL-GP	B16-GP	KPC-GP
Gata2		B-ALL-GP	B16-GP	KPC-GP
Gatad2a		B-ALL-GP	B16-GP	KPC-GP
Gatad2b		B-ALL-GP	B16-GP	KPC-GP
Hdgf		B-ALL-GP	B16-GP	KPC-GP
Hhex	LCMV	B-ALL-GP	B16-GP	KPC-GP
Hmgb2		B-ALL-GP	B16-GP	KPC-GP
Hmgxb4		B-ALL-GP	B16-GP	KPC-GP
Jun		B-ALL-GP	B16-GP	KPC-GP
Klf2	LCMV	B-ALL-GP	B16-GP	KPC-GP
Klf4	LCMV	B-ALL-GP	B16-GP	KPC-GP
mKO2	LCMV	B-ALL-GP	B16-GP	KPC-GP
Mlxipl	LCMV	B-ALL-GP	B16-GP	KPC-GP
Morc3		B-ALL-GP	B16-GP	KPC-GP
Mxd3	LCMV	B-ALL-GP	B16-GP	KPC-GP
Myc	LCMV	B-ALL-GP	B16-GP	KPC-GP
Nanog	LCMV	B-ALL-GP	B16-GP	KPC-GP
Nfil3	LCMV	B-ALL-GP	B16-GP	KPC-GP
NGFR		B-ALL-GP	B16-GP	KPC-GP
Nr0b1		B-ALL-GP	B16-GP	KPC-GP

Nr1h3	LCMV	B-ALL-GP	B16-GP	KPC-GP
Nr3c1	LCMV	B-ALL-GP	B16-GP	KPC-GP
Nrf1		B-ALL-GP	B16-GP	KPC-GP
Pax5	LCMV	B-ALL-GP	B16-GP	KPC-GP
Pou2f2	LCMV	B-ALL-GP	B16-GP	KPC-GP
Pou5f1	LCMV	B-ALL-GP	B16-GP	KPC-GP
Ppara	LCMV	B-ALL-GP	B16-GP	KPC-GP
Ppard	LCMV	B-ALL-GP	B16-GP	KPC-GP
Pparg	LCMV	B-ALL-GP	B16-GP	KPC-GP
Prdm1	LCMV	B-ALL-GP	B16-GP	KPC-GP
Rara		B-ALL-GP	B16-GP	KPC-GP
Rarb		B-ALL-GP	B16-GP	KPC-GP
Rarg		B-ALL-GP	B16-GP	KPC-GP
Runx1	LCMV	B-ALL-GP	B16-GP	KPC-GP
Runx2		B-ALL-GP	B16-GP	KPC-GP
Runx3	LCMV	B-ALL-GP	B16-GP	KPC-GP
Rxra	LCMV	B-ALL-GP	B16-GP	KPC-GP
Satb1	LCMV	B-ALL-GP	B16-GP	KPC-GP
Sox2	LCMV	B-ALL-GP	B16-GP	KPC-GP
Sox5	LCMV	B-ALL-GP	B16-GP	KPC-GP
Spi1	LCMV	B-ALL-GP	B16-GP	KPC-GP
Tbx21	LCMV	B-ALL-GP	B16-GP	KPC-GP
Tcf7	LCMV	B-ALL-GP	B16-GP	KPC-GP
Tfap4	LCMV	B-ALL-GP	B16-GP	KPC-GP
Thy1.1	LCMV	B-ALL-GP	B16-GP	KPC-GP
Tox	LCMV	B-ALL-GP	B16-GP	KPC-GP
Trp53	LCMV	B-ALL-GP	B16-GP	KPC-GP
Yy1		B-ALL-GP	B16-GP	KPC-GP
Zbtb7b	LCMV	B-ALL-GP	B16-GP	KPC-GP
Zfp281		B-ALL-GP	B16-GP	KPC-GP
Znf143		B-ALL-GP	B16-GP	KPC-GP
Znf296		B-ALL-GP	B16-GP	KPC-GP
Znf512		B-ALL-GP	B16-GP	KPC-GP
Zscan12	LCMV	B-ALL-GP	B16-GP	KPC-GP

Table S1. List of retroviral vectors utilized in the scGOF-seq screen. Gene expressed and the screen/disease setting the gene was included in is noted. For all these vectors, the vector was purchased from Vector Builder, the genes are mouse-derived, except for DTR and mKO2, and have EGFP as the reporter.

Table S2

**Primer**	**Step**	**Sequence**	**Final Molarity**
EGFP Rev 1	Reverse Transcription	CTCGATGTTGTGGCGGATCT	0.5 – 1 µM
FWD for cDNA	cDNA Amplification (ORF only)	CTACACGACGCTCTTCCGAT*C*T	0.2 µM
EGFP Rev for cDNA	cDNA Amplification (GEX + ORF)	ACACCTTGCCGATGTCG*A*G	0.06 µM
EGFP Rev PCR	cDNA Amplification (ORF only)	AAGTCGATGCCCTTCAGC*T*C	0.2 µM
CommonFWD	Nested PCR - ORF Outer, Middle, and Inner	GATCTACACTCTTTCCCTACACGACGC	0.3 µM
EGFP Rev 2	Nested PCR - ORF Outer	CTTGTAGTTGCCGTCGTCCT	0.5 µM
EGFP Rev 3	Nested PCR - ORF Inner	GAACTTCAGGGTCAGCTTGC	0.5 µM

Table S2. Primers for custom amplification of ORF libraries. Primers and concentrations used for custom cDNA amplification for ORF library. Which step in the amplification process the custom primer is used is also listed.

Table S3

**FASTQ Prefix**	**Library Type**
GEX	Standard gene expression
CMO	CellPlex multiplexing feature barcode
**ORF_NoPCR**	**ORF without nested PCR enrichment**
ORF_2xPCR	ORF with standard nested PCR enrichment (outer+inner)
ORF	ORF with standard nested PCR enrichment (outer+inner)

Table S3. FASTQ Prefix File Names. Guide to the FASTQ file name prefixes and the library types to which they correspond.
